# Systematic Review of Platelet-Rich Plasma in Medical and Surgical Specialties: Quality, Evaluation, Evidence, and Enforcement

**DOI:** 10.3390/jcm13154571

**Published:** 2024-08-05

**Authors:** Eqram Rahman, Parinitha Rao, Hany Niamey Abu-Farsakh, Chirag Thonse, Imran Ali, Alice E. Upton, Shwetha Y. Baratikkae, Jean D. A. Carruthers, Ash Mosahebi, Nima Heidari, William Richard Webb

**Affiliations:** 1 Research and Innovation Hub, Innovation Aesthetics, London WC2H 9JQ, UKw.r.webb1@icloud.com (W.R.W.); 2 The Skin Address, Aesthetic Dermatology Practice, Bengaluru 560080, India; 3 Istishari Hospital, Amman 840431, Jordan; 4 Manipal Hospital, Millers Road, Bengaluru 560052, India; 5 Integrated Medical Centre, Crawford Street, London WIU 6BE, UK; 6 Jansithaa Fertility Centre & Hospital, Bengaluru 560079, India; 7 Department of Ophthalmology, University of British Columbia, Vancouver, BC V6T 1Z4, Canada; 8 Department of Plastic and Reconstructive Surgery, Royal Free Hospital, Hampstead NW3 2QG, UK; amosahebi@gmail.com; 9 Medical Supercomputation and Machine Learning, European Quantum Medical, London E10 5NP, UK; 10 Foot, Ankle and Limb Reconstruction, Orthopaedic Surgeon, London W1G 7ET, UK; 11 Pharmacy, Science and Technology, George Emil Palade University of Medicine, 540142 Targu Mures, Romania

**Keywords:** platelet-rich plasma, PRP, quality control, regenerative medicine, WESS-PQR, efficacy, regulatory standards

## Abstract

**Background:** Platelet-rich plasma (PRP) is widely used in various medical and surgical specialties for its regenerative properties, including aesthetics (facial rejuvenation, hair restoration, and skin tightening) and orthopedics (treatment of tendinitis and osteoarthritis). However, the inconsistent literature on PRP’s efficacy and safety leads to critical knowledge gaps. This systematic review evaluates quality control measures in PRP preparation and application and explores the regulatory environment governing its clinical use. **Methods:** Following PRISMA guidelines, a comprehensive search was conducted across multiple databases, including PubMed, EMBASE, and Web of Science, for studies published from January 2020 to April 2024. The review included randomized controlled trials (RCTs) involving human participants undergoing PRP treatment for aesthetic or regenerative purposes. Key parameters such as the PRP preparation methods, platelet concentration, and quality control measures were analyzed. The study protocol was registered with PROSPERO (ID: CRD42024557669). **Results:** Out of 75 RCTs involving 5726 patients, the review identified significant variability in PRP preparation methods and application techniques, including differences in centrifugation protocols and platelet concentration levels. A new evidence-based scoring system, the William–Eqram Scoring System for PRP Quality Reporting (WESS-PQR), was proposed to address these inconsistencies. Correlation analysis revealed a strong positive correlation (r = 0.79) between proper temperature control during preparation and PRP efficacy. Initial platelet count assessment showed a moderate positive correlation (r = 0.57) with efficacy. **Conclusions:** Standardized PRP preparation protocols and robust regulatory frameworks are urgently needed to ensure the safety and efficacy of PRP treatments. The proposed WESS-PQR scoring system can serve as a valuable tool for clinicians and researchers, promoting consistency and reliability in PRP applications.

## 1. Introduction

First identified by Ferrari in 1987, an autologous transfusion component post-open-heart surgery as an alternative to homologous blood product transfusion [1], namely platelet-rich plasma (PRP), is defined as autologous plasma with a platelet concentration significantly higher than the baseline range of 150,000 platelets/μL to 450,000 platelets/μL [2], typically reaching levels four to seven times greater [3,4]. PRP is enriched with Vascular Endothelial Growth Factor (VEGF), Platelet-Derived Growth Factor (PDGF), Transforming Growth Factor-beta (TGF-β), Epidermal Growth Factor (EGF), Insulin-Like Growth Factor (IGF), and Cytokines essential for tissue repair and regeneration [5,6,7]. 

PRP is widely used in different medical and surgical specialties with increasing interest in minimally invasive aesthetics and orthopedics. In aesthetic applications, it is commonly employed for facial rejuvenation [8,9,10,11,12,13], hair restoration [14,15,16,17,18,19,20,21,22,23], and skin tightening [24], leveraging its ability to stimulate collagen production and improve skin texture and tone. In regenerative orthopedics, PRP is utilized to treat musculoskeletal conditions such as tendinitis, tendinopathies, osteoarthritis [25,26,27,28,29,30,31,32,33,34], and ligament injuries, facilitating faster recovery and improved healing outcomes [6,25,26,27,28,29,30,31,32,33,34,35,36,37,38,39,40,41,42,43,44,45,46,47,48,49,50,51,52,53,54,55,56,57,58,59,60,61,62,63,64,65,66]. Its applications also extend to oral and maxilla-facial surgery [67,68,69,70,71,72], wound healing [70,73,74,75,76], cardiovascular repair [77,78,79], and infertility [80,81,82,83]. 

Despite its growing popularity, several critical knowledge gaps necessitate a systematic review of PRP in both aesthetic and regenerative medicine. The existing literature on PRP efficacy and safety is extensive but often inconsistent, with studies reporting varying outcomes. This variability is reflected in multiple studies that show diverse results, making it challenging to draw definitive conclusions about the effectiveness and safety of PRP treatments. Notably, some studies highlight significant improvements in clinical outcomes [11,23,28,30,34,35,36,38,39,41,42,45,46,50], while others present more modest or negligible benefits [52,54,55,56,57,59,60,64,65,66,77,84,85]. 

Further complicating the assessment are differences in study designs, patient populations, and PRP preparation methods. These factors contribute to the inconsistent findings across the literature, underscoring the need for more standardized approaches in PRP research. Several investigations emphasize the importance of standardized PRP protocols to ensure reproducibility and the reliability of results [86,87,88,89,90,91,92,93,94,95,96,97,98,99,100,101,102,103,104]. 

Moreover, the lack of uniform reporting standards has led to difficulties in comparing and synthesizing data from different studies. Despite the promising potential of PRP, the heterogeneity in existing research calls for a concerted effort to establish clear guidelines and reporting standards to enhance the comparability of future studies [105,106,107,108,109,110,111].

The existing literature on PRP efficacy and safety is extensive but often inconsistent, with studies reporting varying outcomes [11,23,28,30,34,35,36,38,39,41,42,45,46,50,52,54,55,56,57,59,60,64,65,66,77,86,87,88,89,90,91,92,93,94,95,96,97,98,99,100,101,102,103,104,105,106,107,108,109,110,111]. Some research highlights significant benefits, while other studies show minimal or no improvement compared to control treatments, making it challenging for clinicians to make evidence-based decisions [60,84,85].

There is wide variability in PRP preparation and application methods, including differences in centrifugation techniques, platelet concentration, and the presence of leukocytes. These variations can impact treatment outcomes, yet, to date, no universally accepted standard for PRP preparation has been employed [7,19,32,43,61,67,95,112,113,114,115,116,117]. Commercial PRP separation systems exhibit significant variability, making it essential to understand their unique advantages to effectively extend their clinical application across a broad range of conditions [32,43,118,119]. More recently, automation of the PRP production has been developed and promoted to standardize production (e.g., Arthrex Angel™ system) [7,118].

Randomized controlled trials (RCTs) on PRP treatments follow strict protocols to assess efficacy and safety, ensuring standardized procedures and controlled environments [11,21,29,90,101,102]. However, the initial scoping review identified significant variability in preparation and application that can significantly impact outcomes. Therefore, it is prudent to explore the quality control measures followed in these studies, including the standardization of PRP preparation methods, consistency of platelet concentrations, and adherence to procedural protocols. 

Quality control in PRP preparation and application is another critical area often overlooked. Inconsistent preparation methods and a lack of standardized protocols can lead to unpredictable results and potential safety issues. Evaluating the current quality control measures and identifying best practices is highly desired for ensuring the reliability and effectiveness of PRP treatments [120].

Additionally, the regulatory landscape for PRP therapies is fragmented and varies widely across different regions. This lack of uniform regulation can affect the approval, commercialization, and clinical application of PRP [113,117]. A comprehensive review of the regulatory environment is required to identify gaps, propose improvements, and the implementation of quality control checks to ensure the safe and effective use of PRP.

The primary aim of this systematic review is to assess the quality control measures in the preparation and application of PRP. This review will examine the standardization efforts in PRP preparation and propose an evidence-based scoring system for PRP Quality Reporting. The secondary objective is to explore the regulatory environment governing the use of PRP in clinical practice. This review will analyze the current regulatory standards, identify gaps and challenges in regulation, and propose recommendations for improving regulatory oversight to include quality control. 

## 2. Method

This systematic review with meta-analyses was undertaken following the Preferred Reporting Items for Systematic Reviews and Meta-Analyses (PRISMA) guidelines [121]. The review undertaken utilized the format in the Cochrane Handbook for Systematic Reviews of Interventions [122]. A concise description of the study protocol was registered with the International Prospective Register of Systematic Reviews (PROSPERO) (https://www.crd.york.ac.uk/prospero/, record ID: CRD42024557669).

### 2.1. Information Source and Search Strategy

The initial search was performed in February 2024 and updated in April 2024. The databases PubMed/MEDLINE (United States National Library of Medicine, Bethesda, MD, USA), EMBASE (Elsevier, Amsterdam, the Netherlands), Chinese Biomedical Literature Database (CBM), Web of Science (Clarivate, Ukraine), China Network Knowledge Information (CNKI), Chinese Science Journal Database (VIP), and Wanfang Database were searched to identify relevant studies. Grey literature was searched for in the System for Information on Grey Literature in Europe OpenGrey (www.opengrey.eu). All the databases were searched from January 2020 to the present (April 2024).

The search strategy comprised the use of free text and index terms such as: “Platelet-Rich Plasma”, “PRP”, “Autologous Platelet Gel”, “Platelet Concentrate”, “Aesthetic Medicine”, “Cosmetic Dermatology”, “Facial Rejuvenation”, “Skin Rejuvenation”, “Hair Restoration”, “Alopecia Treatment”, “Anti-Aging”, “Dermal Fillers”, “Regenerative Medicine”, “Tissue Regeneration”, “Wound Healing”, “Osteoarthritis Treatment”, “Surgical procedures”, “Tendon Repair”, “Ligament Healing”, “Cartilage Repair”, “Musculoskeletal Injuries”, “infertility”, “clinical medicine”, “Efficacy”, “Safety”, “Adverse Effects”, “Quality Control”, “Standardization”, “Regulatory Approval”, and “Observational Studies”. Boolean operators (AND, OR) were used to enhance the search. (Supplemental Material: S1, which demonstrates the PubMed, EMBASE, and Web of Science search strategy). The reference citations of all retrieved articles were manually reviewed to identify additional publications.

### 2.2. Study Selection Criteria

Studies were included if they met the eligibility criteria described using the PICOS framework. For the patient population (P), studies involving human participants undergoing PRP treatment for aesthetic or regenerative purposes were selected. Regarding intervention (I), PRP had to be the primary intervention, including various preparation methods and application techniques. In terms of control (C), comparisons to placebo, no treatment, or other standard treatments such as hyaluronic acid fillers or corticosteroids were considered.

The outcomes (O) of interest included quality control measures focused on the standardization of PRP preparation methods, consistency of platelet concentration, and adherence to procedural protocols in the randomized controlled trials. 

The study design (S) strictly considered randomized controlled trials published in English. Studies exclusively focused on a specific subset of applications or procedures without broader relevance to the review’s scope were excluded. Studies that did not follow the CONSORT guideline or did not provide information in line with the CONSORT checklist were also excluded.

Two authors, ER and WW, independently screened the titles and abstracts of identified studies and removed duplicates. The studies were then exported to the EndNote Reference Library software version 20.0.1 (Clarivate Analytics, Philadelphia, PA, USA) for further management. Next, the articles were thoroughly evaluated to determine whether they met the predefined inclusion criteria. Full texts of potentially useful articles were reviewed in their entirety. Any discrepancies and disagreements were addressed and resolved by the third author, PR.

### 2.3. Outcome of Interest and Outcome Measure

The main outcome of interest was to explore the efficacy reporting of the RCTs in different medical and surgical indications and their correlation to the quality reporting in,

I. Laboratory Tests

Pre-treatment laboratory tests, including a platelet count, to ensure the patient has an adequate platelet level for effective PRP preparation.

II. Standardization of PRP Preparation Methods

Assessing whether studies followed consistent protocols for PRP preparation, including centrifugation protocols [4], final platelet concentration, platelet activation methods, and growth factor concentration reporting.

III. Temperature control during preparation, and adherence to aseptic techniques

### 2.4. Data Extraction

For each identified study, relevant data were extracted using a standardized data extraction form. This form captured essential details, including the study characteristics, participant demographics (participant number and condition treated), specifics of the PRP intervention (preparation method and platelet concentration), and efficacy reporting. Extracted data were organized and managed using EndNote and Microsoft Excel or Google Sheets for data organization and preliminary analyses.

### 2.5. Quality Assessment of Studies

The risk of bias in each included study was independently evaluated by two authors (ER and WW) employing the Cochrane risk-of-bias tool for randomized trials (RoB 2). Any discrepancies were resolved by a third author (PR). RoB 2 is designed to address specific aspects of trial design, conduct, and reporting, structured into five domains: bias arising from the randomization process, bias due to deviations from intended interventions, bias due to missing outcome data, bias in the measurement of the outcome, and bias in the selection of the reported result. Each domain contains a set of signaling questions that authors must objectively judge based on the content of the studies. Once these questions are answered, a risk-of-bias judgment is made, categorizing each domain into one of three levels: low risk of bias, some concerns, or high risk of bias.

### 2.6. Proposed Scoring System for the PRP Quality Reporting

We propose the William–Eqram Scoring System for PRP Quality Reporting (WESS-PQR) to address the significant inconsistencies and lack of standardization in PRP preparation and reporting across clinical studies. This comprehensive scoring system evaluates seven critical criteria: initial platelet count assessment, centrifugation protocol, final platelet concentration, platelet activation method, growth factor concentration reporting, temperature control during preparation, and adherence to aseptic techniques. Each criterion is scored on a scale from 0 to 5, with higher scores indicating better adherence to quality standards. The total score ranges from 0 to 35 points, categorized as follows:

- Range 30–35 points: Very good adherence to evidence-based practices;
- Range 25–29 points: Good adherence with some minor issues;
- Range 20–24 points: Fair adherence with several notable gaps;
- Range 15–19 points: Poor adherence with significant issues;
- Range 0–14 points: Very poor adherence, significant risk of suboptimal PRP quality (Table 1).

### 2.7. Statistical Analysis

Due to the high heterogeneity and variability of the data identified in our scoping review, performing a meta-analysis was not feasible. The studies varied significantly in terms of PRP preparation methods, application techniques, and outcome measures, leading to substantial differences in results that could not be reliably combined.

However, we conducted a correlation analysis to investigate the relationship between PRP Quality Reporting and its efficacy. Specifically, we examined how well-reported quality control measures in PRP preparation (such as initial platelet count, centrifugation protocols, final platelet concentration, and growth factor concentration) correlated with reported efficacy outcomes in the included studies.

All quality reporting metrics were expressed in percentages to standardize the data and facilitate comparison across studies. To visualize the results of this correlation analysis, we generated a heat map. This heat map highlights the strength and direction of correlations between various quality reporting criteria and efficacy outcomes, providing a clear visual representation of the relationships observed. The heat map was created using Python’s seaborn library (Waskom, 2021), ensuring that the analysis was both rigorous and reproducible [123].

To ensure the reliability and applicability of the WESS-PQR, we conducted a rigorous validation process. The validation involved several steps:

1. Selection of Studies for Validation: A subset of 20 studies was randomly selected from the included studies to represent a range of PRP preparation and reporting quality;
2. Independent Scoring: Three independent reviewers, blinded to each other’s scores, assessed the selected studies using the WESS-PQR criteria. Each reviewer assigned scores for the seven criteria based on the information provided in the studies;
3. Inter-Rater Reliability: The consistency of the scores among the three reviewers was evaluated using Cohen’s kappa coefficient (κ). A κ value above 0.75 indicates excellent agreement, between 0.60 and 0.75 indicates good agreement, and below 0.60 indicates fair to poor agreement;
4. Statistical Analysis: The average scores for each criterion and the total scores were calculated. The variability in scores was analyzed using standard deviation (SD) and coefficient of variation (CV).

Statistical analyses were conducted using the STATA 18 software (StataCorp. 2023. Stata Statistical Software: Release 18. College Station, TX, USA: StataCorp LLC). Statistical significance was defined as *p* < 0.05.

### 2.8. Patient and Public Involvement

There was no patient or public involvement in the design or reviewing process.

### 2.9. Deviation from the Protocol

There was no deviation from the protocol.

## 3. Result

### 3.1. Study Selection Process

**Figure 1:**
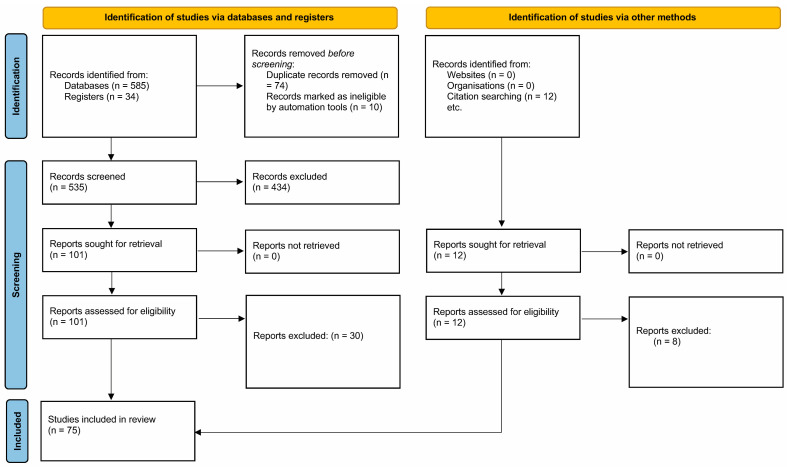
PRISMA flow diagram. The PRISMA checklist has also been included as Supplemental Material—S2 to ensure adherence to reporting guidelines.

In the initial search, 585 and 34 records were retrieved from electronic databases and clinical trial registers, respectively. Additionally, 12 records were identified through citation searching. After removing 84 records using the automatic tool and manual de-duplication, 547 records were examined with titles, abstracts, and interventions. Subsequently, 91 records from databases, 10 records from registers, and 12 from the citation searching underwent full-text review. Finally, 75 studies were eligible for data extraction and quantitative analysis (Figure 1).

### 3.2. Characteristics of the Included Studies

The systematic review included a total of 75 randomized controlled trials (RCTs) involving 5726 participants, covering a diverse range of medical conditions across various subspecialties (Table 2).

#### 3.2.1. Orthopedics and Musculoskeletal Conditions

The bulk of studies focused on orthopedics and musculoskeletal conditions. Several RCTs addressed knee osteoarthritis (17) [35,37,38,40,41,42,45,46,47,50,55,56,57,59,60,62,66] assessing the impact of PRP injections on pain reduction, joint function, and cartilage regeneration. Other joint and tendon conditions studied include ankle osteoarthritis (2) [53,54], hip osteoarthritis (1) [52], Achilles tendon rupture and tendinopathy (3) [26,31,33], rotator cuff tendinopathy (1) [28], lateral elbow tendinopathy (2) [97,127], plantar fasciitis (2) [100,132], glenohumeral osteoarthritis (1) [48], lumbar herniated disc (2) [108,109], lumbar discogenic pain (1) [111], lumbar facet joint disease (1) [129], low back pain (3) [99,131,145], myofascial pain syndrome (1) [86], and carpal ligament release (1) [106]. These studies explored PRP’s efficacy in promoting healing, reducing pain, and improving functional outcomes.

#### 3.2.2. Dermatology and Hair Restoration

The review included studies on dermatology and hair restoration, specifically addressing androgenetic alopecia (9) [21,23,87,92,103,107,124,128,141], alopecia areata (1) [139], and chronic telogen effluvium (1) [89]. The other dermatologic conditions studied were facial skin rejuvenation (1) [136] and inflammatory acne vulgaris (1) [98], evaluating PRP’s benefits in enhancing skin quality and tissue regeneration.

#### 3.2.3. Wound Healing

PRP’s role in wound healing was another significant focus. RCTs investigated PRP in treating chronic refractory wounds (1) [85], sacrococcygeal pilonidal sinus (2) [88,91], and diabetic foot ulcers (1) [104], aiming to assess PRP’s effectiveness in accelerating wound healing and reducing recurrence rates. 

#### 3.2.4. Oral and Maxillofacial Conditions

Several studies focused on oral and maxillofacial conditions, including extraction socket healing (1) [96], dental implant stability (1), dental implant placement (1) [133], gingival recession (1) [110], gingiva depigmentation (1) [94], and orthodontic tooth movement (1) [138].

#### 3.2.5. Ocular Conditions

Two RCTs examined the use of PRP in treating dry eye disease (2) [95,130], assessing its impact on tear production and overall eye health. Another study focused on macular holes (1) [143], exploring PRP’s potential in promoting retinal healing and improving visual outcomes.

#### 3.2.6. Reproductive Health

The review included studies on reproductive health conditions such as Asherman syndrome (1) [134], thin endometrium (1) [126], and repeated implantation failure (1) [135].

#### 3.2.7. Miscellaneous Conditions

Other medical conditions studied included oral lichen planus (1) [93], leprosy trophic ulcers (1) [144], hemophilic knee arthritis (1) [39], and urinary anabolic metabolites (1) [137]. These trials explored PRP’s therapeutic benefits in pain management, namely promoting healing and improving functional outcomes across diverse clinical scenarios.

### 3.3. Outcome of Interest and Outcome Measures

#### 3.3.1. Initial Platelet Count Assessment

Out of the 75 studies, 35 (46.6%) assessed the initial platelet count before PRP preparation. This assessment is vital to ensure that the baseline platelet levels are adequate for achieving a therapeutic concentration after centrifugation. The remaining 40 studies did not report any initial platelet count assessment (53.4%).

#### 3.3.2. Final Platelet Concentration

The final platelet concentration in PRP was reported in 24 out of the 75 studies (32%). These studies generally aimed for a platelet concentration of 3 to 7 times the baseline. For instance, Ye et al. (2024) achieved a 3× to 6× baseline platelet concentration [64] and Efendieva et al. (2023) achieved a 4× baseline concentration [126]. However, most studies failed to report the final platelet concentration (68%). 

#### 3.3.3. Centrifugation Protocols

Centrifugation is a pivotal step in PRP preparation, influencing the concentration and quality of the final PRP product. The reviewed studies demonstrated a wide range of centrifugation protocols, categorized into single-spin and double-spin methods; 27 studies utilized a single-spin protocol (36%), whilst 31 studies employed a double-spin protocol (41.3%) and 17 did not report anything (22.7%). 

Only five studies explicitly reported the RCF used in their centrifugation protocols (6.7%), highlighting a significant gap in standardized reporting with seventy studies failing to report RCF (93.3%). The minimum RPM reported was 500 rpm by Huang et al. (2022) for a single spin lasting 8 min [46], while the maximum RPM was 3800 rpm reported by Navani et al. (2024) with the first spin lasting 1.5 min and the second spin lasting 5 min [99]. The shortest spin duration was 3 min (Kang et al., 2023) [95] and the longest was 30 min (Metheetrairut, 2022) [130].

#### 3.3.4. Platelet Activation Methods

Only 14 out of the 75 studies reported using platelet activation methods (18.7%), such as calcium chloride or thrombin. Conversely, 81.3% did not report any activation method, which can impede growth factor release and the overall therapeutic outcomes.

#### 3.3.5. Growth Factor Concentration Reporting

Growth factor concentration, a critical component of PRP’s therapeutic potential, was reported in only 5 studies (6.7%) and 70 studies failed to report growth factor concentrations (93.3%). This lack of reporting limits the ability to correlate clinical outcomes with specific growth factor levels and hinders the understanding of PRP’s mechanism of action.

#### 3.3.6. Temperature Control during Preparation

Temperature control during PRP preparation was mentioned in 19 studies (25.3%), with the remaining 56 studies failing to report temperature control (74.7%). Temperature management is essential to maintain platelet viability and functionality. Studies like those by Efendieva et al. (2023) [126] and Keene et al. (2022) [31] highlighted the importance of maintaining consistent temperatures to preserve the quality of PRP.

#### 3.3.7. Adherence to Aseptic Techniques

Adherence to aseptic techniques was reported in 65 studies (86.7%), emphasizing the importance of sterility in PRP preparation and administration to prevent infections and ensure patient safety. Conversely, 10 studies (13.3%) did not report aseptic technique adherence. Studies consistently following aseptic protocols are likely to produce more reliable and reproducible outcomes.

### 3.4. Quality Assessment of the Included Studies

In the domain of bias arising from the randomization process, 74% of the studies were assessed as having a low risk, reflecting the use of rigorous and clearly reported randomization methods. However, 19% had some concerns due to insufficient detail on randomization and 7% were rated as high risk due to inadequate or poorly described processes.

For bias due to deviations from the intended intervention, 70% of the studies were judged to have a low risk, indicating close adherence to intervention protocols. Conversely, 23% had some concerns due to minor deviations or incomplete reporting and 7% were assessed as high risk because of significant deviations impacting study outcomes.

In assessing bias due to missing outcome data, 67% of the studies had a low risk, demonstrating adequate management and reporting. However, 29% had some concerns due to incomplete handling of missing data and 4% were rated as high risk due to substantial unaddressed missing data.

Regarding bias in the measurement of outcomes, 68% of the studies were at low risk, ensuring accurate and consistent measurement techniques. Meanwhile, 21% had some concerns due to inconsistencies or incomplete reporting and 11% were assessed as high risk due to significant issues in outcome measurement.

In the domain of bias in the selection of reported results, 73% of the studies were judged to have a low risk, indicating comprehensive and unbiased reporting. However, 20% had some concerns due to potential selective reporting and 7% were rated as high risk due to clear evidence of selective reporting.

**Figure 2:**
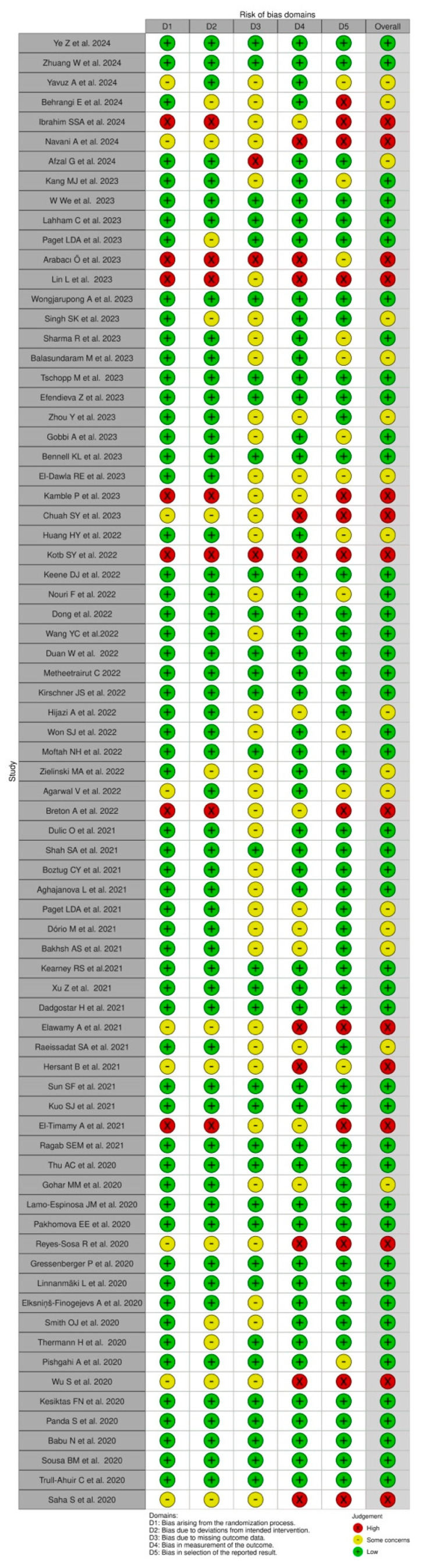
Risk of bias of the included studies (Traffic Light plot) [21,28,30,31,33,35,37,38,39,40,41,42,45,46,47,48,50,52,53,54,55,56,57,59,60,62,64,65,66,82,85,86,87,88,89,91,92,93,94,95,96,97,98,99,100,103,104,105,107,108,109,110,111,124,125,126,127,128,129,130,131,132,133,134,135,136,137,138,139,140,141,142,143,144].

**Figure 3:**
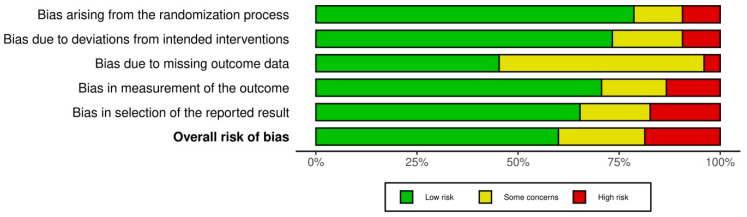
Summary of the Risk of bias.

Overall, 65% of the included studies were judged to have a low risk of bias across most domains, providing reasonable confidence in their findings. In contrast, 25% had some concerns, primarily due to incomplete or unclear reporting on randomization and intervention protocols, and 10% were assessed as high risk, largely due to significant methodological flaws such as inadequate randomization, missing outcome data, and selective reporting (Table 3, Figure 2 and Figure 3).

### 3.5. Statistical Analysis

#### 3.5.1. Validation Result of the WESS-PQR

Cohen’s kappa coefficient for the seven criteria ranged from 0.72 to 0.82, indicating good to excellent agreement among the reviewers. The detailed validation statistics are presented in Table 4. 

**Figure 4:**
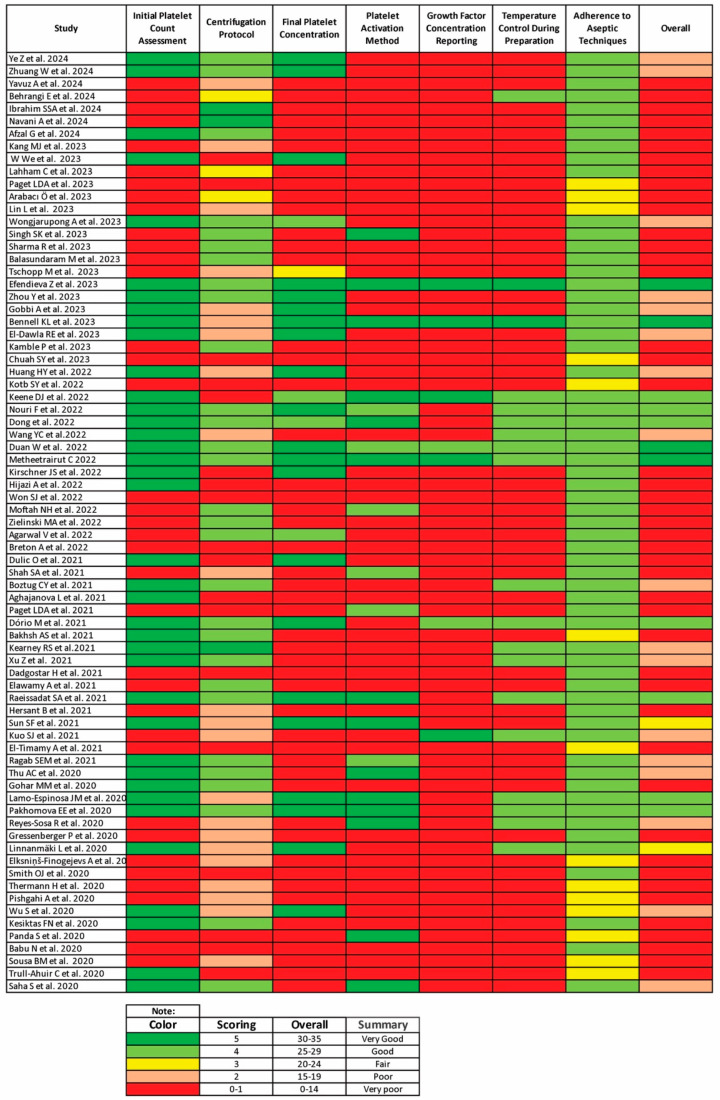
The assessment of the included studies using WESS-PQR [21,28,30,31,33,35,37,38,39,40,41,42,45,46,47,48,50,52,53,54,55,56,57,59,60,62,64,65,66,82,85,86,87,88,89,91,92,93,94,95,96,97,98,99,100,103,104,105,107,108,109,110,111,124,125,126,127,128,129,130,131,132,133,134,135,136,137,138,139,140,141,142,143,144].

The assessment of the included studies using WESS-PQR is presented in Figure 4.

#### 3.5.2. Correlation Analysis

The heatmap suggests a strong positive correlation between quality control measures (such as initial platelet count, centrifugation protocol, final platelet concentration, platelet activation method, growth factor concentration reporting, temperature control during preparation, and adherence to aseptic techniques) and PRP efficacy. This indicates that meticulous quality control significantly impacts PRP treatment outcomes.

For example, initial platelet count assessment shows a moderate positive correlation (0.57) with efficacy, suggesting that higher initial platelet counts are associated with improved PRP efficacy. Final platelet concentration demonstrates a strong positive correlation (0.67) with efficacy, suggesting that achieving higher final platelet concentrations is crucial for effective PRP treatments. Temperature control during preparation shows a strong positive correlation (0.79) with efficacy, indicating that maintaining proper temperature control during preparation is vital for PRP efficacy.

**Figure 5:**
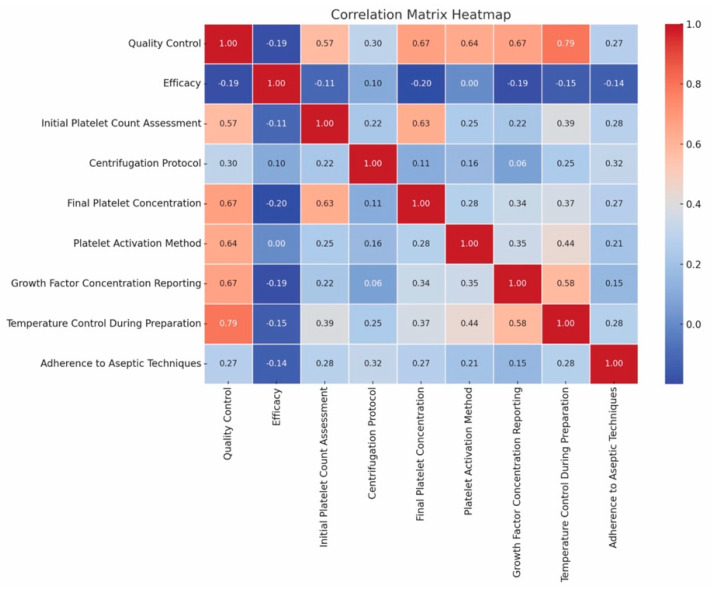
Correlation matrix heatmap demonstrating a strong and statistically significant relationship between quality control measures and PRP efficacy.

While the overall regression model has a *p*-value (Prob > F-statistic) of 0.356, indicating it is not statistically significant, the individual correlations remain strong (Figure 5).

## 4. Discussion

The use of PRP across multiple medical disciplines has increased rapidly over the past 20 years [146]. However, our study is the largest systematic review of the RCTs across multiple specialties, highlighting the lack of scientific rigor and understanding, i.e., basic and fundamental measurements are not recorded. The studies have shown that over 50% of the studies failed to report baseline platelet counts prior to PRP processing. This is a significant failure due to the wide range of values for healthy platelet counts being 100,000 platelets/μL to 450,000 platelets/μL [2]. Furthermore, failure to report post-production platelet counts was identified in 68% of the included studies in this study. The failure to report pre- and post-production platelet numbers prevents the study from reporting the concentration values of the administered PRP. The absence of this crucial data raises significant concerns about the concentration of PRP administered and its potential impact on outcomes and efficacy. This information should be considered the minimum requirement for quality control. The lack of such data highlights a broader issue—the insufficient understanding and implementation of quality control systems within healthcare and medicine.

The analysis of the included studies also revealed that the majority reported centrifugation speeds in terms of rpm. However, rpm values alone cannot be standardized due to variations in rotor sizes and designs across different centrifuge models. This inconsistency in reporting centrifugation speeds leads to significant variability in the applied centrifugal forces during PRP preparation, which can impact the reproducibility and comparability of study outcomes. For precise standardization and reproducibility, it is essential to report Relative Centrifugal Force (RCF) instead of rpm [147].

RCF is a measure of the actual force exerted on the blood components during centrifugation and is expressed in units of gravity (g). It provides a consistent and standardized metric and considers both the rpm and the radius of the rotor. 

The formula to calculate *RCF* is

*RCF =* 1.118 × 10^−5^ × R * (RPM)^2^ * RPM

where R is the radius of the rotor reported in centimeters. By both calculating and reporting RCF, researchers ensure that the centrifugation process can be accurately replicated across different studies, regardless of the centrifuge model used [147]. This standardization is critical because even small variations in the centrifugal force can significantly affect the separation of platelets from other blood components, thereby impacting the quality and efficacy of PRP preparation. The calculation and reporting of RCF should therefore be mandated in PRP research to enhance the reliability, reproducibility, and comparability of the results across different studies and clinical settings [147].

Another crucial aspect of PRP preparation that significantly impacts clinical outcomes is the repeated centrifugation of lysed platelets, inducing the release of their growth factors and other bioactive contents. Studies have shown that lysing the platelets through a second centrifugation step enhances the release of growth factors such as PDGF, TGF-β, and VEGF [148]. This elevated release of growth factors correlates with improved clinical outcomes, including faster healing and better tissue regeneration [149]. For instance, Amable et al. (2013) demonstrated that repeated centrifugation to lyse platelets significantly increased the concentration of growth factors in PRP, leading to superior clinical results in tissue regeneration applications [112].

Although various PRP kits were utilized across the studies, this does not necessarily guarantee the quality of the PRP produced. The effectiveness and reliability of PRP treatments depend on multiple factors beyond the kit itself, such as the centrifugation protocol, platelet concentration, activation methods, and adherence to standardized procedures. The variability in these factors across different studies suggests that simply using a commercial PRP kit is insufficient to ensure the consistency and quality of the final product. RCF values attributed to centrifugation for standardization were only reported in 6.7% of the included studies and 93.3% failed to report as a scientific standard. Mazzocca et al. (2012) emphasized the importance of quality control in PRP preparation, showing that standardized preparation protocols in RCTs resulted in higher platelet concentrations and better clinical outcomes compared to the non-standardized methods commonly reported in observational studies [115]. The findings from Pietrzak and Eppley (2005) highlighted the critical importance of platelet concentration in PRP’s therapeutic efficacy [150]. Their study reveals a dose–response relationship between platelet concentration and the release of key growth factors such as PDGF and TGF-β. Specifically, they found that PRP with higher platelet counts—up to five times the baseline—resulted in significantly elevated levels of these growth factors, which are essential for wound healing and tissue regeneration [150]. 

A critical issue identified in this review is the lack of initial assessment of the patient’s platelet count before PRP preparation in many studies. According to FDA guidelines (Title 21 CFR Part 640.21) [151], it is essential to assess and monitor the donor’s platelet count to ensure that it meets the required thresholds before plateletpheresis begins. Specifically, the donor’s platelet count should be at least 150,000 platelets/μL before starting the procedure. Failure to perform this initial assessment can lead to suboptimal platelet concentrations in the PRP, thereby affecting its efficacy. The omission of this critical step in many studies underscores the need for adherence to regulatory guidelines to ensure the quality and effectiveness of PRP treatments.

Quality assurance in PRP therapy is not solely dependent on the technical aspects of preparation and administration. The operator’s expertise plays a critical role in ensuring the correct implementation of protocols. Inadequate training and variability in practitioner skills can introduce another layer of inconsistency, affecting the therapeutic outcomes of PRP. Thus, establishing stringent training programs and certification requirements for practitioners is essential to maintain high standards of practice.

**Figure 6:**
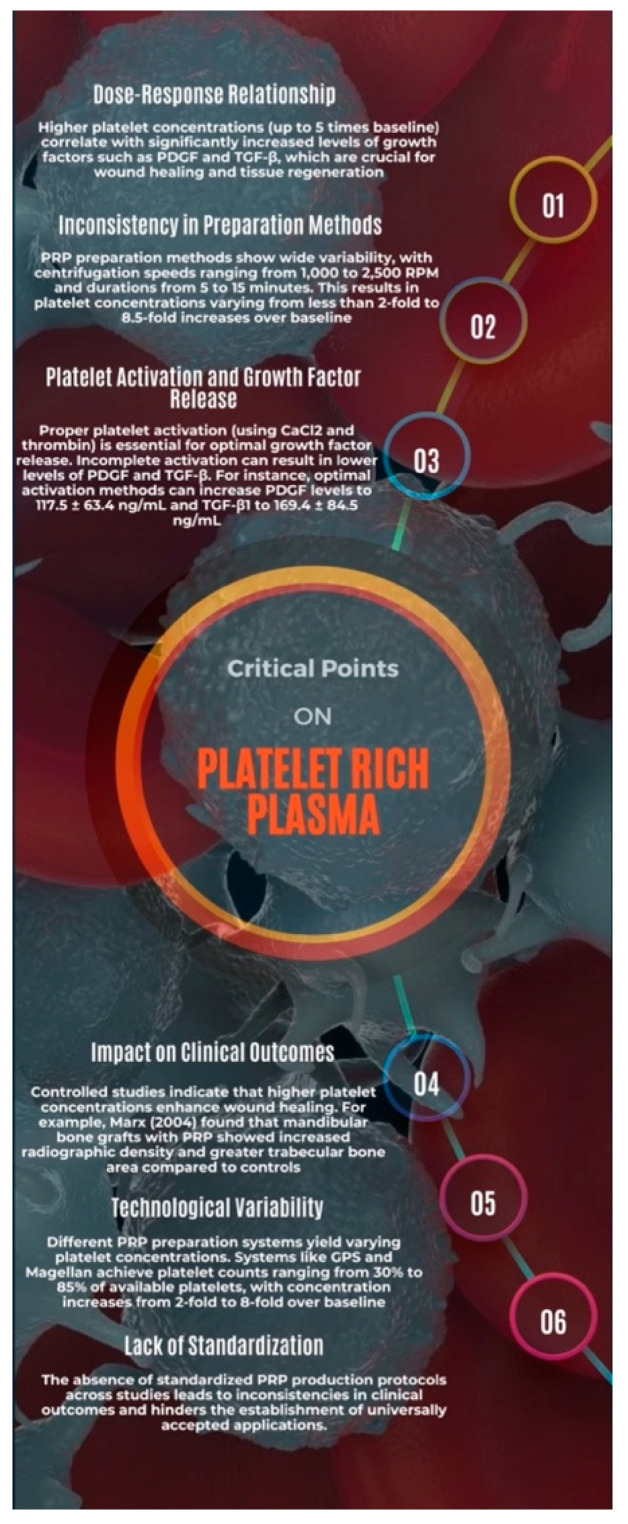
Infographics of the critical point on PRP preparation [3,152,153].

Additionally, the lack of standardized quality control measures across different regions and clinical settings can lead to significant disparities in PRP therapy outcomes. This variation calls for the development of universal guidelines that can be adopted globally to harmonize practices. Such guidelines should be based on evidence from robust clinical studies and expert consensus to cover all aspects of PRP therapy, including patient selection, preparation protocols, and post-procedural care (Figure 6).

The International Cellular Medical Society (ICMS) has set forth comprehensive guidelines for PRP usage, stressing standardized preparation techniques, rigorous patient selection, and adherence to procedural protocols [154]. However, the adoption and implementation of these guidelines are uneven, leading to significant inconsistencies in PRP practices. The lack of a unified approach across different regions results in a fragmented regulatory landscape, which ultimately compromises the quality of PRP treatments and may be a major contributing factor to outcome reporting.

The FDA’s regulation of PRP under the Code of Federal Regulations Title 21 also mandates stringent criteria for donor eligibility, blood collection, and processing. These regulations emphasize maintaining proper platelet counts and ensuring controlled temperatures during the separation process. While the FDA guidelines are robust, the enforcement and compliance across various clinical settings are inconsistent. This variability can result in suboptimal PRP preparations, directly affecting clinical outcomes and patient safety. The absence of stringent oversight mechanisms exacerbates this issue, leaving gaps that can be exploited by less scrupulous practitioners.

European regulations, governed by directives such as 2002/98/EC and 2005/62/EC, require adherence to Good Manufacturing Practices (GMP) for blood and blood components, including PRP [155]. These directives are intended to ensure all steps, from collection to administration and are conducted under strict quality control conditions. However, the enforcement of these standards is inconsistent across different member states. This inconsistency not only leads to variability in PRP quality but also undermines the trust in regulatory frameworks that are supposed to safeguard patient health. The lack of a harmonized enforcement strategy within Europe reveals a significant regulatory shortfall.

In the United Arab Emirates, the Dubai Health Authority (DHA) has established detailed guidelines that mandate licensing for healthcare facilities and certification for practitioners offering PRP treatments [156]. These guidelines emphasize using standardized and approved equipment and maintaining aseptic techniques. Despite these comprehensive regulations, the uniformity in enforcement remains questionable. 

The regulatory landscape in the United Kingdom, under the Medicines and Healthcare Products Regulatory Agency (MHRA), reveals a significant gap: failure to implement strict guidelines and quality control checks in the manufacture and use of PRP in clinical settings. The absence of specific national guidelines for PRP therapy under the MHRA leads to considerable inconsistencies in PRP application and quality. This regulatory void allows for a wide range of practices, some of which may be suboptimal or even unsafe. The lack of clear standardized guidelines not only affects patient outcomes but also undermines the credibility of PRP therapies. These gaps highlight an urgent need for the MHRA to develop and implement comprehensive guidelines to regulate PRP treatments effectively. Given the paucity of clear regulatory guidelines, it was not possible to ascertain whether the included studies met the geographic-specific regulatory requirements and this again may contribute to the lack of consensus in the use of PRP. 

This study boasts several strengths. Firstly, it provides a comprehensive evaluation of PRP preparation and reporting standards across a large number of RCTs, encompassing a wide range of medical and surgical subspecialties. This broad scope allows for a thorough understanding of current practices and highlights areas requiring standardization. Secondly, the development and validation of the WESS-PQR is a significant contribution; this robust scoring system enhances the quality and transparency of PRP research by providing a standardized framework for assessing and reporting PRP preparation methods.

However, the study also has some limitations. Firstly, the variability in study design, PRP preparation protocols, and outcome measures affected the comparability and generalizability of the findings. Secondly, due to the high variability in PRP preparation methods, a meta-analysis was not feasible. We used qualitative synthesis and recommended more standardized study designs for future meta-analyses, which would help in achieving reproducibility and reliability of results. Thirdly, the potential for publication bias cannot be overlooked. Studies with positive or significant results are more likely to be published, while those with negative or null findings may be underrepresented, skewing the overall assessment. Similar trends were observed in the recent systematic reviews [157,158,159,160,161]. Despite these limitations, we used multiple databases and employed independent reviewers to screen and select studies based on predefined criteria. This minimizes selection bias and ensures a comprehensive unbiased inclusion of relevant studies. This methodological rigor supports the reliability of our findings on PRP preparation and reporting quality.

For patients, the findings of this review highlight the importance of receiving PRP treatments from practitioners who adhere to standardized protocols and comply with regulatory guidelines. Patients should be aware of the variability in PRP preparation and seek treatment from certified and well-trained practitioners to ensure the highest quality and efficacy of PRP therapy. Understanding the role of regulatory guidelines can also empower patients to make informed decisions about their treatment options.

For practitioners, this review underscores the critical need for adherence to standardized preparation and application protocols. Practitioners must ensure that they follow established guidelines to maintain the quality and safety of PRP treatments. Additionally, practitioners should stay informed about the latest regulatory requirements and quality control measures to enhance their practice and optimize patient outcomes. The review also highlights the importance of thorough training and certification programs to reduce variability in PRP practices.

Future research should focus on several key areas to address the gaps identified in this review. First, there is a need for large-scale multicentre randomized controlled trials (RCTs) to validate the findings from observational studies and case series. These trials should adhere to standardized PRP preparation and application protocols to provide robust evidence on the efficacy and safety of PRP treatments. Additionally, future studies should include detailed assessments of long-term outcomes to understand the sustained benefits and potential risks associated with PRP therapy.

Future research should explore the molecular mechanisms underlying PRP’s therapeutic effects to optimize its use in various clinical applications. Investigating the impact of different preparation techniques on the release of growth factors and cytokines can provide insights into enhancing the efficacy of PRP.

Furthermore, there is a need for the development and validation of universal guidelines for PRP therapy. Comparative studies across different regulatory frameworks can help identify best practices and inform the creation of harmonized standards. Lastly, research should focus on the implementation and enforcement of regulatory guidelines to ensure that they are effectively integrated into clinical practice, thereby improving the consistency and reliability of PRP treatments globally.

## 5. Conclusions

The lack of standardized preparation protocols and uneven enforcement of regulatory standards severely compromise the reliability and efficacy of PRP treatments and suboptimal patient outcomes and may be a major factor in a wide variety of outcomes reported in similar studies. To realize the full potential of PRP therapy, there is an urgent need for unified enforceable standards to ensure safety, efficacy, and consistency such as the currently proposed WESS-PQR. Only through such rigorous standardization can we truly harness the regenerative promise of PRP and deliver its benefits to patients worldwide, ensuring the highest standards of care and outcomes.

## Figures and Tables

**Table 1:** William–Eqram Scoring System for PRP Quality Reporting (WESS-PQR).

Criterion	0 Points	1 Point	2 Points	3 Points	4 Points	5 Points
**Initial Platelet Count Assessment**	No assessment	Poorly documented	Partially assessed or documented	Assessed but not fully documented	Assessed with minor gaps	Fully assessed and documented as per FDA guidelines
**Centrifugation Protocol**	No protocol described	Non-standard protocol	Single spin mentioning rpm and/or g only	Single spin mentioning RCF	Double spin mentioning rpm and/or g only	Double spin mentioning RCF
**Final Platelet Concentration**	Not reported or below baseline	Less than 1-time above baseline	1–2 times above baseline	2–3 times above baseline	3–4 times above baseline	>4 times above baseline
**Platelet Activation Method**	No activation method described	Poorly described activation method	Alternative activation method, partially documented	Alternative activation method, fully documented	Activation with CaCl_2_ and thrombin, partially documented	Activation with CaCl_2_ and thrombin, fully documented
**Growth Factor Concentration Reporting**	No reporting of growth factors	Minimal documentation of growth factors	Inconsistent or poorly documented growth factor levels	Limited reporting of growth factors	Reporting of key growth factors but with some gaps	Comprehensive reporting of growth factors (e.g., PDGF, TGF-β) with ELISA
**Temperature Control During Preparation**	No mention of temperature control	Minimal or no temperature control	Poor documentation of temperature control	Temperature control documented but inconsistently applied	Temperature controlled but with minor deviations	Temperature strictly controlled within 20–24 °C throughout the process
**Adherence to Aseptic Techniques**	No adherence to aseptic techniques	Minimal adherence to aseptic techniques	Poor documentation of aseptic techniques	General adherence but with notable documentation gaps	Adherence to aseptic techniques with minor documentation gaps	Strict adherence to aseptic techniques fully documented

**Table 2:** Characteristics of the included studies.

First Author et al.	Year	Type of Study	Study Area	Number of Patients	Efficacy Reporting	Summary of the PRP Quality Reporting
Ye Z et al. [64]	2024	RCT	ACL Reconstruction	120	Not Effective	Poor
Zhuang W et al. [66]	2024	RCT	Knee Osteoarthritis	120	Effective	Poor
Yavuz A et al. [110]	2024	RCT	Gingival Recession	12	Inconclusive	Very poor
Behrangi E et al. [87]	2024	RCT	Androgenetic Alopecia	60	Inconclusive	Very poor
Ibrahim SSA et al. [94]	2024	RCT	Gingiva Depigmentation	10	Effective	Very poor
Navani A et al. [99]	2024	RCT	Chronic Low Back Pain	57	Effective	Very poor
Afzal G et al. [124]	2024	RCT	Androgenetic Alopecia	27	Effective	Very poor
Kang MJ et al. [95]	2023	RCT	Dry eye	36	Inconclusive	Very poor
W Wei et al. [107]	2023	RCT	Androgenetic Alopecia	30	Effective	Very poor
Lahham C et al. [96]	2023	RCT	Extraction Socket Healing	20	Inconclusive	Very poor
Paget LDA et al. [53]	2023	RCT	Ankle Osteoarthritis	100	Not Effective	Very poor
Arabacı Ö et al. [125]	2023	RCT	Meningomyelocele sac repair	20	Effective	Very poor
Lin L et al. [85]	2023	RCT	Chronic Refractory Wounds	120	Effective	Very poor
Wongjarupong A et al. [108]	2023	RCT	Lumbar herniated disc	30	Effective	Poor
Singh SK et al. [103]	2023	RCT	Androgenetic alopecia	80	Effective	Very poor
Sharma R et al. [100]	2023	RCT	Plantar Fasciitis	90	Effective	Very poor
Balasundaram M et al. [21]	2023	RCT	Androgenetic alopecia	64	Inconclusive	Very poor
Tschopp M et al. [60]	2023	RCT	Knee Osteoarthritis	99	Not Effective	Very poor
Efendieva Z et al. [126]	2023	RCT	Thin Endometrium	115	Effective	Excellent
Zhou Y et al. [65]	2023	RCT	Knee cartilage lesions	60	Inconclusive	Poor
Gobbi A et al. [45]	2023	RCT	Knee osteoarthritis	50	Not Effective	Poor
Bennell KL et al. [35]	2023	RCT	Knee Osteoarthritis	288	Not Effective	Excellent
El-Dawla RE et al. [89]	2023	RCT	Chronic telogen effluvium	30	Inconclusive	Poor
Kamble P et al. [127]	2023	RCT	Lateral Elbow Tendinopathy	65	Effective	Very poor
Chuah SY et al. [128]	2023	RCT	Androgenetic alopecia	50	Effective	Very poor
Huang HY et al. [46]	2022	RCT	Knee Osteoarthritis	95	Inconclusive	Poor
Kotb SY et al. [129]	2022	RCT	Lumbar Facet Joint Disease	30	Effective	Very poor
Keene DJ et al. [31]	2022	RCT	Achilles Tendon Rupture	230	Not Effective	Good
Nouri F et al. [52]	2022	RCT	Hip osteoarthritis	105	Effective	Good
Dong C et al. [37]	2022	RCT	Knee Osteoarthritis	77	Inconclusive	Good
Wang YC et al. [62]	2022	RCT	Knee Osteoarthritis	116	Inconclusive	Poor
Duan W et al. [39]	2022	RCT	Hemophilic Knee Arthritis	190	Not Effective	Excellent
Metheetrairut C et al. [130]	2022	RCT	Dry eye disease	10	Inconclusive	Excellent
Kirschner JS et al. [48]	2022	RCT	Glenohumeral osteoarthritis	70	Not Effective	Very poor
Hijazi A et al. [93]	2022	RCT	Oral lichen planus	20	Inconclusive	Very poor
Won SJ et al. [131]	2022	RCT	Low back pain	34	Effective	Very poor
Moftah NH et al. [98]	2022	RCT	Inflammatory acne vulgaris	30	Effective	Very poor
Zielinski MA et al. [111]	2022	RCT	Lumbar Discogenic Pain	26	Not Effective	Very poor
Agarwal V et al. [86]	2022	RCT	Myofascial Pain Syndrome	30	Inconclusive	Very poor
Breton A et al. [132]	2022	RCT	Plantar Fasciitis	50	Effective	Very poor
Dulic O et al. [40]	2021	RCT	Knee osteoarthritis	195	Effective	Very poor
Shah SA et al. [133]	2021	RCT	Dental Implant	84	Effective	Very poor
Boztug CY et al. [88]	2021	RCT	Pilonidal sinus	49	Effective	Poor
Aghajanova L et al. [134]	2021	RCT	Asherman syndrome	10	Not Effective	Very poor
Paget LDA et al. [54]	2021	RCT	Ankle Osteoarthritis	100	Not Effective	Very poor
Dório M et al. [38]	2021	RCT	Knee Osteoarthritis	62	Not Effective	Good
Bakhsh AS et al. [135]	2021	RCT	Repeated implantation failure	100	Effective	Very poor
Kearney RS et al. [30]	2021	RCT	Achilles Tendinopathy	240	Not Effective	Poor
Xu Z et al. [109]	2021	RCT	Lumbar Disc Herniation	132	Inconclusive	Poor
Dadgostar H et al. [28]	2021	RCT	Rotator Cuff Tendinopathy	58	Inconclusive	Very poor
Elawamy A et al. [41]	2021	RCT	Chronic Knee Osteoarthritis	200	Not Effective	Very poor
Raeissadat SA et al. [56]	2021	RCT	Knee Osteoarthritis	238	Effective	Good
Hersant B et al. [136]	2021	RCT	Facial Skin Rejuvenation	93	Inconclusive	Very poor
Sun SF et al. [59]	2021	RCT	Knee osteoarthritis	85	Inconclusive	Fair
Kuo SJ et al. [137]	2021	RCT	Urinary anabolic metabolites	24	Effective	Poor
El-Timamy A et al. [138]	2021	RCT	Orthodontic tooth movement	16	Inconclusive	Very poor
Ragab SEM et al. [139]	2021	RCT	Alopecia areata	60	Inconclusive	Poor
Thu AC et al. [140]	2020	RCT	Adhesive capsulitis	64	Inconclusive	Poor
Gohar MM et al. [91]	2020	RCT	Sacrococcygeal pilonidal sinus	120	Effective	Very poor
Lamo-Espinosa JM et al. [50]	2020	RCT	Knee osteoarthritis	60	Inconclusive	Good
Pakhomova EE et al. [141]	2020	RCT	Androgenetic Alopecia	69	Inconclusive	Good
Reyes-Sosa R et al. [57]	2020	RCT	Knee Osteoarthritis	60	Effective	Poor
Gressenberger P et al. [92]	2020	RCT	Androgenetic Alopecia	30	Not Effective	Very poor
Linnanmäki L et al. [97]	2020	RCT	Lateral Epicondylitis	119	Not Effective	Fair
Elksniņš-Finogejevs A et al. [42]	2020	RCT	Knee Osteoarthritis	40	Inconclusive	Very poor
Smith OJ et al. [104]	2020	RCT	Diabetic Foot Ulcers	18	Inconclusive	Very poor
Thermann H et al. [33]	2020	RCT	Achilles tendinopathy	36	Not Effective	Very poor
Pishgahi A et al. [55]	2020	RCT	Knee Osteoarthritis	92	Inconclusive	Very poor
Wu S et al. [142]	2020	RCT	Reconstruction of posterior cruciate ligament	58	Effective	Poor
Kesiktas FN et al. [47]	2020	RCT	Knee Osteoarthritis	54	Inconclusive	Very poor
Panda S et al. [82]	2020	RCT	Periodontal pockets	26	Inconclusive	Very poor
Babu N et al. [143]	2020	RCT	Macular holes	60	Effective	Very poor
Sousa BM et al. [105]	2020	RCT	Temporomandibular Joint Disorders	80	Effective	Very poor
Trull-Ahuir C et al. [106]	2020	RCT	Carpal Ligament Release	50	Effective	Very poor
Saha S et al. [144]	2020	RCT	Leprosy Trophic Ulcer	118	Effective	Poor

**Table 3:** Risk of bias of the included studies.

**Study**	**Bias Arising from the Randomization Process**	**Bias Due to Deviation from the Intended Intervention**	**Bias Due to Missing Outcome Data**	**Bias in the Measurement of Outcome**	**Bias in the Selection of the Reported Result**	**Overall**
Ye Z et al., 2024 [64]	Low	Low	Low	Low	Low	Low
Zhuang W et al., 2024 [66]	Low	Low	Low	Low	Low	Low
Yavuz A et al., 2024 [110]	Some concerns	Low	Some concerns	Low	Some concerns	Some concerns
Behrangi E et al., 2024 [87]	Low	Some concerns	Some concerns	Low	High	Some concerns
Ibrahim SSA et al., 2024 [94]	High	High	Some concerns	Some concerns	High	High
Navani A et al., 2024 [99]	Some concerns	Some concerns	Some concerns	High	High	High
Afzal G et al., 2024 [124]	Low	Low	High	Low	Low	Some concerns
Kang MJ et al., 2023 [95]	Low	Low	Some concerns	Low	Some concerns	Low
W Wei et al., 2023 [107]	Low	Low	Low	Low	Low	Low
Lahham C et al., 2023 [96]	Low	Low	Low	Low	Low	Low
Paget LDA et al., 2023 [53,54]	Low	Some concerns	Low	Low	Low	Low
Arabacı Ö et al., 2023 [125]	High	High	High	High	Some concerns	High
Lin L et al., 2023 [85]	High	High	Some concerns	High	High	High
Wongjarupong A et al., 2023 [108]	Low	Low	Low	Low	Low	Low
Singh SK et al., 2023 [103]	Low	Some concerns	Some concerns	Low	Low	Some concerns
Sharma R et al., 2023 [100]	Low	Low	Some concerns	Low	Some concerns	Low
Balasundaram M et al., 2023 [21]	Low	Low	Some concerns	Low	Some concerns	Some concerns
Tschopp M et al., 2023 [60]	Low	Low	Low	Low	Low	Low
Efendieva Z et al., 2023 [126]	Low	Low	Low	Low	Low	Low
Zhou Y et al., 2023 [65]	Low	Low	Some concerns	Some concerns	Low	Some concerns
Gobbi A et al., 2023 [45]	Low	Low	Some concerns	Low	Some concerns	Low
Bennell KL et al., 2023 [35]	Low	Low	Low	Low	Low	Low
El-Dawla RE et al., 2023 [89]	Low	Low	Some concerns	Some concerns	Some concerns	Some concerns
Kamble P et al., 2023 [127]	High	High	Some concerns	Some concerns	High	High
Chuah SY et al., 2023 [128]	Some concerns	Some concerns	Some concerns	High	High	High
Huang HY et al., 2022 [46]	Low	Low	Some concerns	Low	Some concerns	Some concerns
Kotb SY et al., 2022 [129]	High	High	High	High	High	High
Keene DJ et al., 2022 [31]	Low	Low	Low	Low	Low	Low
Nouri F et al., 2022 [52]	Low	Low	Some concerns	Low	Some concerns	Low
Dong et al., 2022 [37]	Low	Low	Low	Low	Low	Low
Wang YC et al., 2022 [62]	Low	Low	Some concerns	Low	Low	Low
Duan W et al., 2022 [39]	Low	Low	Low	Low	Low	Low
Metheetrairut C 2022 [130]	Low	Low	Low	Low	Low	Low
Kirschner JS et al., 2022 [48]	Low	Low	Low	Low	Low	Low
Hijazi A et al., 2022 [93]	Low	Low	Some concerns	Some concerns	Low	Some concerns
Won SJ et al., 2022 [131]	Low	Low	Some concerns	Low	Some concerns	Low
Moftah NH et al., 2022 [98]	Low	Low	Low	Low	Low	Low
Zielinski MA et al., 2022 [111]	Low	Some concerns	Some concerns	Low	Low	Some concerns
Agarwal V et al., 2022 [86]	Some concerns	Low	Some concerns	Low	Some concerns	Some concerns
Breton A et al., 2022 [132]	High	High	Some concerns	Some concerns	High	High
Dulic O et al., 2021 [40]	Low	Low	Some concerns	Low	Low	Low
Shah SA et al., 2021 [133]	Low	Low	Low	Low	Low	Low
Boztug CY et al., 2021 [88]	Low	Low	Some concerns	Low	Low	Low
Aghajanova L et al., 2021 [134]	Low	Low	Some concerns	Low	Low	Low
Paget LDA et al., 2021	Low	Low	Some concerns	Some concerns	Low	Some concerns
Dório M et al., 2021 [54]	Low	Low	Some concerns	Some concerns	Low	Some concerns
Bakhsh AS et al., 2021 [135]	Low	Low	Some concerns	Some concerns	Low	Some concerns
Kearney RS et al., 2021 [30]	Low	Low	Low	Low	Low	Low
Xu Z et al., 2021 [109]	Low	Low	Low	Low	Low	Low
Dadgostar H et al., 2021 [28]	Low	Low	Low	Low	Low	Low
Elawamy A et al., 2021 [41]	Some concerns	Some concerns	Some concerns	High	High	High
Raeissadat SA et al., 2021 [56]	Low	Low	Some concerns	Some concerns	Low	Some concerns
Hersant B et al., 2021 [136]	Some concerns	Some concerns	Some concerns	High	Some concerns	High
Sun SF et al., 2021 [59]	Low	Low	Low	Low	Low	Low
Kuo SJ et al., 2021 [137]	Low	Low	Low	Low	Low	Low
El-Timamy A et al., 2021 [138]	High	High	Some concerns	Some concerns	High	High
Ragab SEM et al., 2021 [139]	Low	Low	Low	Low	Low	Low
Thu AC et al., 2020 [140]	Low	Low	Low	Low	Low	Low
Gohar MM et al., 2020 [91]	Low	Low	Some concerns	Some concerns	Low	Some concerns
Lamo-Espinosa JM et al., 2020 [50]	Low	Low	Low	Low	Low	Low
Pakhomova EE et al., 2020 [141]	Low	Low	Low	Low	Low	Low
Reyes-Sosa R et al., 2020 [57]	Some concerns	Some concerns	Some concerns	High	High	High
Gressenberger P et al., 2020 [92]	Low	Low	Low	Low	Low	Low
Linnanmäki L et al., 2020 [97]	Low	Low	Low	Low	Low	Low
Elksniņš-Finogejevs A et al., 2020 [42]	Low	Low	Some concerns	Low	Low	Low
Smith OJ et al., 2020 [104]	Low	Some concerns	Some concerns	Low	Low	Low
Thermann H et al., 2023 [33]	Low	Some concerns	Low	Low	Low	Low
Pishgahi A et al., 2020 [55]	Low	Low	Low	Low	Some concerns	Low
Wu S et al., 2020 [142]	Some concerns	Some concerns	Some concerns	High	High	High
Kesiktas FN et al., 2020 [47]	Low	Low	Low	Low	Low	Low
Panda S et al., 2020 [82]	Low	Low	Low	Low	Low	Low
Babu N et al., 2020 [143]	Low	Low	Low	Low	Low	Low
Sousa BM et al., 2020 [105]	Low	Low	Low	Low	Low	Low
Trull-Ahuir C et al., 2020 [106]	Low	Low	Low	Low	Low	Low
Saha S et al., 2020 [144]	Some concerns	Some concerns	Some concerns	High	High	High

**Table 4:** Validation statistics of the William–Eqram Scoring System for PRP Quality Reporting (WESS-PQR).

Criterion	Cohen’s Kappa (κ)	Average Score	Standard Deviation (SD)	Coefficient of Variation (CV)
Initial Platelet Count Assessment	0.75	3.5	0.8	22.9%
Centrifugation Protocol	0.78	3.2	1.0	31.3%
Final Platelet Concentration	0.80	3.8	0.6	15.8%
Platelet Activation Method	0.72	3.0	1.2	40.0%
Growth Factor Concentration Reporting	0.76	2.9	1.1	37.9%
Temperature Control During Preparation	0.82	3.7	0.7	18.9%
Adherence to Aseptic Techniques	0.77	4.0	0.5	12.5%
**Total Score**	-	24.1	3.4	14.1%

## Data Availability

Data supporting the findings of this study are available from the corresponding author upon reasonable request.

## Supplementary Materials

The following supporting information can be downloaded at https://www.mdpi.com/article/10.3390/jcm13154571/s1. Supplementary Material S1: Search strategy. Supplementary Material S2: PRISMA checklist

## References

[1] Moscicka P., Przylipiak A. (2021). History of autologous platelet-rich plasma: A short review. J. Cosmet. Dermatol..

[2] Giles C. (1981). The platelet count and mean platelet volume. Br. J. Haematol..

[3] Marx R.E. (2001). Platelet-rich plasma (PRP): What is PRP and what is not PRP?. Implant. Dent..

[4] Alves R., Grimalt R. (2018). A Review of Platelet-Rich Plasma: History, Biology, Mechanism of Action, and Classification. Skin. Appendage Disord..

[5] Marck R.E., Gardien K.L.M., Vlig M., Breederveld R.S., Middelkoop E. (2019). Growth Factor Quantification of Platelet-Rich Plasma in Burn Patients Compared to Matched Healthy Volunteers. Int. J. Mol. Sci..

[6] Yung Y.L., Fu S.C., Cheuk Y.C., Qin L., Ong M.T., Chan K.M., Yung P.S. (2017). Optimisation of platelet concentrates therapy: Composition, localisation, and duration of action. Asia Pac. J. Sports Med. Arthrosc. Rehabil. Technol..

[7] Sundman E.A., Cole B.J., Fortier L.A. (2011). Growth factor and catabolic cytokine concentrations are influenced by the cellular composition of platelet-rich plasma. Am. J. Sports Med..

[8] Buzalaf M.A.R., Levy F.M. (2022). Autologous platelet concentrates for facial rejuvenation. J. Appl. Oral Sci..

[9] Neiva-Sousa M., Carracha C., Nunes da Silva L., Valejo Coelho P. (2023). Does Platelet-Rich Plasma Promote Facial Rejuvenation? Revising the Latest Evidence in a Narrative Review. J. Cutan. Aesthet. Surg..

[10] Banihashemi M., Zabolinejad N., Salehi M., Hamidi Alamdari D., Nakhaizadeh S. (2021). Platelet-rich Plasma use for facial rejuvenation: A clinical trial and review of current literature. Acta Biomed..

[11] Alam M., Hughart R., Champlain A., Geisler A., Paghdal K., Whiting D., Hammel J.A., Maisel A., Rapcan M.J., West D.P. (2018). Effect of Platelet-Rich Plasma Injection for Rejuvenation of Photoaged Facial Skin: A Randomized Clinical Trial. JAMA Dermatol..

[12] Peng G.L. (2019). Platelet-Rich Plasma for Skin Rejuvenation: Facts, Fiction, and Pearls for Practice. Facial Plast. Surg. Clin. N. Am..

[13] Araco A. (2019). A prospective study comparing topic platelet-rich plasma vs. placebo on reducing superficial perioral wrinkles and restore dermal matrix. J. Cosmet. Laser Ther..

[14] Ramadan W.M., Hassan A.M., Ismail M.A., El Attar Y.A. (2021). Evaluation of adding platelet-rich plasma to combined medical therapy in androgenetic alopecia. J. Cosmet. Dermatol..

[15] Roohaninasab M., Goodarzi A., Ghassemi M., Sadeghzadeh-Bazargan A., Behrangi E., Najar Nobari N. (2021). Systematic review of platelet-rich plasma in treating alopecia: Focusing on efficacy, safety, and therapeutic durability. Dermatol. Ther..

[16] Muhammad A., Iftikhar N., Mashhood A., Saleem Z., Sundus M., Khalid A.A., Khan S., Naveed S., Shahid W., Ajmal U. (2022). Comparison of Efficacy of Platelet-Rich Plasma (PRP) With PRP Microneedling in Androgenetic Alopecia. Cureus.

[17] Maletic A., Dumic-Cule I., Brlek P., Zic R., Primorac D. (2022). Autologous Platelet-Rich Plasma (PRP) for Treating Androgenetic Alopecia: A Novel Treatment Protocol Standardized on 2 Cases. J. Clin. Med..

[18] Mercuri S.R., Paolino G., Di Nicola M.R., Vollono L. (2021). Investigating the Safety and Efficacy of Platelet-Rich Plasma (PRP) Treatment for Female Androgenetic Alopecia: Review of the Literature. Medicina.

[19] Kramer M.E., Keaney T.C. (2018). Systematic review of platelet-rich plasma (PRP) preparation and composition for the treatment of androgenetic alopecia. J. Cosmet. Dermatol..

[20] de Oliveira A.F.Q., Arcanjo F.P.N., Rodrigues M.R.P., Rosa E.S.A.A., Hall P.R. (2023). Use of autologous platelet-rich plasma in androgenetic alopecia in women: A systematic review and meta-analysis. J. Dermatol. Treat..

[21] Balasundaram M., Kumari R., Ramassamy S. (2023). Efficacy of autologous platelet-rich plasma therapy versus topical Minoxidil in men with moderate androgenetic alopecia: A randomized open-label trial. J. Dermatol. Treat..

[22] Borowiecka J.M., Dalewski B., Pałka Ł. (2023). Effectiveness of Platelet-Rich Plasma in the Treatment of Androgenic Alopecia Compared to Placebo and Topical Minoxidil: A Systematic Review. Sci. Pharm..

[23] Rodrigues B.L., Montalvao S.A.L., Cancela R.B.B., Silva F.A.R., Urban A., Huber S.C., Junior J., Lana J., Annichinno-Bizzacchi J.M. (2019). Treatment of male pattern alopecia with platelet-rich plasma: A double-blind controlled study with analysis of platelet number and growth factor levels. J. Am. Acad. Dermatol..

[24] Petit L., Pierard G.E. (2003). Skin-lightening products revisited. Int. J. Cosmet. Sci..

[25] Bowman K.F., Muller B., Middleton K., Fink C., Harner C.D., Fu F.H. (2013). Progression of patellar tendinitis following treatment with platelet-rich plasma: Case reports. Knee Surg. Sports Traumatol. Arthrosc..

[26] Abate M., Di Carlo L., Salini V. (2021). To evaluate the outcomes of PRP treatment in Achilles tendinopathy: An intriguing methodological problem. Orthop. Traumatol. Surg. Res..

[27] Arthur Vithran D.T., Xie W., Opoku M., Essien A.E., He M., Li Y. (2023). The Efficacy of Platelet-Rich Plasma Injection Therapy in the Treatment of Patients with Achilles Tendinopathy: A Systematic Review and Meta-Analysis. J. Clin. Med..

[28] Dadgostar H., Fahimipour F., Pahlevan Sabagh A., Arasteh P., Razi M. (2021). Corticosteroids or platelet-rich plasma injections for rotator cuff tendinopathy: A randomized clinical trial study. J. Orthop. Surg. Res..

[29] Filardo G., Kon E., Di Matteo B., Di Martino A., Tesei G., Pelotti P., Cenacchi A., Marcacci M. (2014). Platelet-rich plasma injections for the treatment of refractory Achilles tendinopathy: Results at 4 years. Blood Transfus..

[30] Kearney R.S., Ji C., Warwick J., Parsons N., Brown J., Harrison P., Young J., Costa M.L., Collaborators A.T.M.T. (2021). Effect of Platelet-Rich Plasma Injection vs Sham Injection on Tendon Dysfunction in Patients with Chronic Midportion Achilles Tendinopathy: A Randomized Clinical Trial. JAMA.

[31] Keene D.J., Alsousou J., Harrison P., O’Connor H.M., Wagland S., Dutton S.J., Hulley P., Lamb S.E., Willett K., PATH-2 Trial Group (2022). Platelet-rich plasma injection for acute Achilles tendon rupture: Two-year follow-up of the PATH-2 randomized, placebo-controlled, superiority trial. Bone Joint J..

[32] Oudelaar B.W., Peerbooms J.C., Huis In‘t Veld R., Vochteloo A.J.H. (2019). Concentrations of Blood Components in Commercial Platelet-Rich Plasma Separation Systems: A Review of the Literature. Am. J. Sports Med..

[33] Thermann H., Fischer R., Gougoulias N., Cipollaro L., Maffulli N. (2023). Endoscopic debridement for non-insertional Achilles tendinopathy with and without platelet-rich plasma. J. Sport. Health Sci..

[34] Wang Y., Han C., Hao J., Ren Y., Wang J. (2019). Efficacy of platelet-rich plasma injections for treating Achilles tendonitis : Systematic review of high-quality randomized controlled trials. Orthopade.

[35] Bennell K.L., Paterson K.L., Metcalf B.R., Duong V., Eyles J., Kasza J., Wang Y., Cicuttini F., Buchbinder R., Forbes A. (2021). Effect of Intra-articular Platelet-Rich Plasma vs Placebo Injection on Pain and Medial Tibial Cartilage Volume in Patients with Knee Osteoarthritis: The RESTORE Randomized Clinical Trial. JAMA.

[36] Bocun L., Jing L., Jia L., Tan Q., Chen J., Huang Z., Guowei C. (2021). Effects of platelet-rich plasma injection for pain control and cartilage repair in knee osteoarthritis: A protocol for the systematic review and meta-analysis of randomized controlled trials in animal models. Medicine.

[37] Dong C., Zhao C., Wang F. (2022). Clinical benefit of high tibial osteotomy combined with the intervention of platelet-rich plasma for severe knee osteoarthritis. J. Orthop. Surg. Res..

[38] Dorio M., Pereira R.M.R., Luz A.G.B., Deveza L.A., de Oliveira R.M., Fuller R. (2021). Efficacy of platelet-rich plasma and plasma for symptomatic treatment of knee osteoarthritis: A double-blinded placebo-controlled randomized clinical trial. BMC Musculoskelet. Disord..

[39] Duan W., Su X., Yu Z., Jiang M., Zhao L., Giannoudis P.V., Guo J.J. (2022). No Benefit to Platelet-rich Plasma Over Placebo Injections in Terms of Pain or Function in Patients with Hemophilic Knee Arthritis: A Randomized Trial. Clin. Orthop. Relat. Res..

[40] Dulic O., Rasovic P., Lalic I., Kecojevic V., Gavrilovic G., Abazovic D., Maric D., Miskulin M., Bumbasirevic M. (2021). Bone Marrow Aspirate Concentrate versus Platelet Rich Plasma or Hyaluronic Acid for the Treatment of Knee Osteoarthritis. Medicina.

[41] Elawamy A., Kamel E.Z., Mahran S.A., Abdellatif H., Hassanien M. (2021). Efficacy of Genicular Nerve Radiofrequency Ablation Versus Intra-Articular Platelet Rich Plasma in Chronic Knee Osteoarthritis: A Single-Blind Randomized Clinical Trial. Pain Physician.

[42] Elksnins-Finogejevs A., Vidal L., Peredistijs A. (2020). Intra-articular platelet-rich plasma vs corticosteroids in the treatment of moderate knee osteoarthritis: A single-center prospective randomized controlled study with a 1-year follow up. J. Orthop. Surg. Res..

[43] Fitzpatrick J., Bulsara M.K., McCrory P.R., Richardson M.D., Zheng M.H. (2017). Analysis of Platelet-Rich Plasma Extraction: Variations in Platelet and Blood Components between 4 Common Commercial Kits. Orthop. J. Sports Med..

[44] Gato-Calvo L., Magalhaes J., Ruiz-Romero C., Blanco F.J., Burguera E.F. (2019). Platelet-rich plasma in osteoarthritis treatment: Review of current evidence. Ther. Adv. Chronic Dis..

[45] Gobbi A., Dallo I., D’Ambrosi R. (2023). Autologous microfragmented adipose tissue and leukocyte-poor platelet-rich plasma combined with hyaluronic acid show comparable clinical outcomes for symptomatic early knee osteoarthritis over a two-year follow-up period: A prospective randomized clinical trial. Eur. J. Orthop. Surg. Traumatol..

[46] Huang H.Y., Hsu C.W., Lin G.C., Lin H.S., Chou Y.J., Liou I.H., Sun S.F. (2022). Comparing efficacy of a single intraarticular injection of platelet-rich plasma (PRP) combined with different hyaluronans for knee osteoarthritis: A randomized-controlled clinical trial. BMC Musculoskelet. Disord..

[47] Kesiktas F.N., Dernek B., Sen E.I., Albayrak H.N., Aydin T., Yildiz M. (2020). Comparison of the short-term results of single-dose intra-articular peptide with hyaluronic acid and platelet-rich plasma injections in knee osteoarthritis: A randomized study. Clin. Rheumatol..

[48] Kirschner J.S., Cheng J., Creighton A., Santiago K., Hurwitz N., Dundas M., Beatty N., Kingsbury D., Konin G., Abutalib Z. (2022). Efficacy of Ultrasound-Guided Glenohumeral Joint Injections of Leukocyte-Poor Platelet-Rich Plasma Versus Hyaluronic Acid in the Treatment of Glenohumeral Osteoarthritis: A Randomized, Double-Blind Controlled Trial. Clin. J. Sport. Med..

[49] Kuffer J., Ziltener J.L. (2022). PRP and knee osteoarthritis. Rev. Med. Suisse.

[50] Lamo-Espinosa J.M., Blanco J.F., Sanchez M., Moreno V., Granero-Molto F., Sanchez-Guijo F., Crespo-Cullel I., Mora G., San Vicente D.D., Pompei-Fernandez O. (2020). Phase II multicenter randomized controlled clinical trial on the efficacy of intra-articular injection of autologous bone marrow mesenchymal stem cells with platelet rich plasma for the treatment of knee osteoarthritis. J. Transl. Med..

[51] Lee J.S., Guo P., Klett K., Hall M., Sinha K., Ravuri S., Huard J., Murphy W.L. (2022). VEGF-attenuated platelet-rich plasma improves therapeutic effect on cartilage repair. Biomater. Sci..

[52] Nouri F., Babaee M., Peydayesh P., Esmaily H., Raeissadat S.A. (2022). Comparison between the effects of ultrasound guided intra-articular injections of platelet-rich plasma (PRP), high molecular weight hyaluronic acid, and their combination in hip osteoarthritis: A randomized clinical trial. BMC Musculoskelet. Disord..

[53] Paget L.D.A., Reurink G., de Vos R.J., Weir A., Moen M.H., Bierma-Zeinstra S.M.A., Stufkens S.A.S., Goedegebuure S., Krips R., Maas M. (2023). Platelet-Rich Plasma Injections for the Treatment of Ankle Osteoarthritis. Am. J. Sports Med..

[54] Paget L.D.A., Reurink G., de Vos R.J., Weir A., Moen M.H., Bierma-Zeinstra S.M.A., Stufkens S.A.S., Kerkhoffs G., Tol J.L., Group P.S. (2021). Effect of Platelet-Rich Plasma Injections vs Placebo on Ankle Symptoms and Function in Patients with Ankle Osteoarthritis: A Randomized Clinical Trial. JAMA.

[55] Pishgahi A., Abolhasan R., Shakouri S.K., Soltani-Zangbar M.S., Dareshiri S., Ranjbar Kiyakalayeh S., Khoeilar A., Zamani M., Motavalli Khiavi F., Pourabbas Kheiraddin B. (2020). Effect of Dextrose Prolotherapy, Platelet Rich Plasma and Autologous Conditioned Serum on Knee Osteoarthritis: A Randomized Clinical Trial. Iran. J. Allergy Asthma Immunol..

[56] Raeissadat S.A., Ghazi Hosseini P., Bahrami M.H., Salman Roghani R., Fathi M., Gharooee Ahangar A., Darvish M. (2021). The comparison effects of intra-articular injection of Platelet Rich Plasma (PRP), Plasma Rich in Growth Factor (PRGF), Hyaluronic Acid (HA), and ozone in knee osteoarthritis; a one year randomized clinical trial. BMC Musculoskelet. Disord..

[57] Reyes-Sosa R., Lugo-Radillo A., Cruz-Santiago L., Garcia-Cruz C.R., Mendoza-Cano O. (2020). Clinical comparison of platelet-rich plasma injection and daily celecoxib administration in the treatment of early knee osteoarthritis: A randomized clinical trial. J. Appl. Biomed..

[58] Shahid A., Malik A., Bukhari A., Shaikh A., Rutherford J., Barkatali B. (2023). Do Platelet-Rich Plasma Injections for Knee Osteoarthritis Work?. Cureus.

[59] Sun S.F., Lin G.C., Hsu C.W., Lin H.S., Liou I.S., Wu S.Y. (2021). Comparing efficacy of intraarticular single crosslinked Hyaluronan (HYAJOINT Plus) and platelet-rich plasma (PRP) versus PRP alone for treating knee osteoarthritis. Sci. Rep..

[60] Tschopp M., Pfirrmann C.W.A., Fucentese S.F., Brunner F., Catanzaro S., Kuhne N., Zwyssig I., Sutter R., Gotschi T., Tanadini M. (2023). A Randomized Trial of Intra-articular Injection Therapy for Knee Osteoarthritis. Investig. Radiol..

[61] Turajane T., Cheeva-Akrapan V., Saengsirinavin P., Lappaiwong W. (2023). Composition of Platelet-Rich Plasma Prepared From Knee Osteoarthritic Patients: Platelets, Leukocytes, and Subtypes of Leukocyte. Cureus.

[62] Wang Y.C., Lee C.L., Chen Y.J., Tien Y.C., Lin S.Y., Chen C.H., Chou P.P., Huang H.T. (2022). Comparing the Efficacy of Intra-Articular Single Platelet-Rich Plasma(PRP) versus Novel Crosslinked Hyaluronic Acid for Early-Stage Knee Osteoarthritis: A Prospective, Double-Blind, Randomized Controlled Trial. Medicina.

[63] Wang Z., Zhu P., Liao B., You H., Cai Y. (2023). Effects and action mechanisms of individual cytokines contained in PRP on osteoarthritis. J. Orthop. Surg. Res..

[64] Ye Z., Chen H., Qiao Y., Wu C., Cho E., Wu X., Li Z., Wu J., Lu S., Xie G. (2024). Intra-Articular Platelet-Rich Plasma Injection After Anterior Cruciate Ligament Reconstruction: A Randomized Clinical Trial. JAMA Netw. Open.

[65] Zhou Y., Li H., Cao S., Han Y., Shao J., Fu Q., Wang B., Wu J., Xiang D., Liu Z. (2023). Clinical Efficacy of Intra-Articular Injection with P-PRP versus that of L-PRP in Treating Knee Cartilage Lesion: A Randomized Controlled Trial. Orthop. Surg..

[66] Zhuang W., Li T., Li Y., Zhang Y., Gao J., Wang X., Ding Q., Li W. (2024). The varying clinical effectiveness of single, three and five intraarticular injections of platelet-rich plasma in knee osteoarthritis. J. Orthop. Surg. Res..

[67] Carlson N.E., Roach R.B. (2002). Platelet-rich plasma: Clinical applications in dentistry. J. Am. Dent. Assoc..

[68] Nikolidakis D., Jansen J.A. (2008). The biology of platelet-rich plasma and its application in oral surgery: Literature review. Tissue Eng. Part. B Rev..

[69] Simonpieri A., Del Corso M., Vervelle A., Jimbo R., Inchingolo F., Sammartino G., Dohan Ehrenfest D.M. (2012). Current knowledge and perspectives for the use of platelet-rich plasma (PRP) and platelet-rich fibrin (PRF) in oral and maxillofacial surgery part 2: Bone graft, implant and reconstructive surgery. Curr. Pharm. Biotechnol..

[70] Menchisheva Y., Mirzakulova U., Yui R. (2019). Use of platelet-rich plasma to facilitate wound healing. Int. Wound J..

[71] Sulistyani L.D., Julia V., Ariawan D., Utomo Y.A., Reksoprodjo M.R., Sandi W.H.S. (2022). Efficacy of Platelet-rich Plasma on Promoting Bone Healing in Maxillofacial Defects: A Systematic Review. J. Int. Dent. Med. Res..

[72] Anitua E., Fernandez-de-Retana S., Alkhraisat M.H. (2021). Platelet rich plasma in oral and maxillofacial surgery from the perspective of composition. Platelets.

[73] Vyas P.U., Khobragade D.S., Mundhada D.R., Shrivastava S.P., Vyas U.B., Pethe A.M. (2023). Preclinical Evaluation of Effi cacy of Processed PRP and Fresh PRP in Diabetic Wound Healing. Int. J. Pharm. Qual. Assur..

[74] Sherrill J.D., Finlay D., Binder R.L., Robinson M.K., Wei X., Tiesman J.P., Flagler M.J., Zhao W., Miller C., Loftus J.M. (2021). Transcriptomic analysis of human skin wound healing and rejuvenation following ablative fractional laser treatment. PLoS ONE.

[75] Verma R., Kumar S., Garg P., Verma Y.K. (2023). Platelet-rich plasma: A comparative and economical therapy for wound healing and tissue regeneration. Cell Tissue Bank..

[76] Pradnyandari N.K.P.D., Natasha R.R. (2022). The role of Platelet-Rich Plasma (PRP) in burn wound healing: A literature-review. Intisari Sains Medis.

[77] Panda S., Purkayastha A., Mohanty R., Nayak R., Satpathy A., Das A.C., Kumar M., Mohanty G., Panda S., Fabbro M.D. (2020). Plasma rich in growth factors (PRGF) in non-surgical periodontal therapy: A randomized clinical trial. Braz. Oral Res..

[78] Jiritano F., Serra R., Nenna A., Curcillo A., Villella F., Nappi F., Chello C., Chello M., Mastroroberto P., Serraino G.F. (2022). Efficacy of prophylactic platelet rich plasma (PRP) following open saphenous vein harvesting in cardiac surgery. Front. Biosci. (Elite Ed.).

[79] Okamura T., Koh E., Yokoyama S. (2001). Effect of autologous platelet-rich plasma (PRP) in cardiac surgery. Kyobu Geka.

[80] Abdullah T.H., Abbas S.H., Al-Obaidi M.T., Abdulraheem Y. (2019). The Efficacy of Platelets Rich Plasma (PRP) for Ovarian Rejuvenation. Indian J. Public. Health Res. Dev..

[81] Barakat E.E., Elsherbeny M.F., Ameen Khalil F.S., EAbdel Raziq H. (2023). Ovarian rejuvenation by PRP (Platelet–Rich Plasma). Benha Med. J..

[82] Panda S.R., Sachan S., Hota S. (2020). A Systematic Review Evaluating the Efficacy of Intra-Ovarian Infusion of Autologous Platelet-Rich Plasma in Patients with Poor Ovarian Reserve or Ovarian Insufficiency. Cureus.

[83] Sabouni R., Tarrab R., Kalaji D., Abbassi H. (2022). A new approach of using platelet-rich autologous plasma to increase the ovarian reservoir in a Syrian patient with ovarian insufficiency: A case report. Ann. Med. Surg..

[84] Frautschi R.S., Hashem A.M., Halasa B., Cakmakoglu C., Zins J.E. (2017). Current Evidence for Clinical Efficacy of Platelet Rich Plasma in Aesthetic Surgery: A Systematic Review. Aesthet. Surg. J..

[85] Lin L., Bi H., Wang X., Shi X. (2023). Impact of Platelet-Rich Fibrin Combined with Silver Nanoparticle Dressing on Healing Time and Therapeutic Efficacy of Chronic Refractory Wounds. Altern. Ther. Health Med..

[86] Agarwal V., Gupta A., Singh H., Kamboj M., Popli H., Saroha S. (2022). Comparative Efficacy of Platelet-Rich Plasma and Dry Needling for Management of Trigger Points in Masseter Muscle in Myofascial Pain Syndrome Patients: A Randomized Controlled Trial. J. Oral Facial Pain Headache.

[87] Behrangi E., Rahimi S.T., Zare S., Goodarzi A., Ghassemi M., Khodadad F., Nouri M., Mozafarpoor S., Dehghani A., Nilforoushzadeh M.A. (2024). Evaluation of the effects of adding an adipose tissue-derived stromal vascular fraction to platelet-rich plasma injection in the treatment of androgenetic alopecia: A randomized clinical trial. Skin Res. Technol..

[88] Boztug C.Y., Karaagac Akyol T., Benlice C., Koc M.A., Doganay Erdogan B., Ozcebe O.I., Kuzu M.A., Akyol C. (2021). Platelet-rich plasma treatment improves postoperative recovery in patients with pilonidal sinus disease: A randomized controlled clinical trial. BMC Surg..

[89] El-Dawla R.E., Abdelhaleem M., Abdelhamed A. (2023). Evaluation of the safety and efficacy of platelet-rich plasma in the treatment of female patients with chronic telogen effluvium: A randomised, controlled, double-blind, pilot clinical trial. Indian J. Dermatol. Venereol. Leprol..

[90] Gentile P., Garcovich S., Bielli A., Scioli M.G., Orlandi A., Cervelli V. (2015). The Effect of Platelet-Rich Plasma in Hair Regrowth: A Randomized Placebo-Controlled Trial. Stem Cells Transl. Med..

[91] Gohar M.M., Ali R.F., Ismail K.A., Ismail T.A., Nosair N.A. (2020). Assessment of the effect of platelet rich plasma on the healing of operated sacrococcygeal pilonidal sinus by lay-open technique: A randomized clinical trial. BMC Surg..

[92] Gressenberger P., Pregartner G., Gary T., Wolf P., Kopera D. (2020). Platelet-rich Plasma for Androgenetic Alopecia Treatment: A Randomized Placebo-controlled Pilot Study. Acta Derm. Venereol..

[93] Hijazi A., Ahmed W., Gaafar S. (2022). Efficacy of intralesional injections of platelet-rich plasma in patients with oral lichen planus: A pilot randomized clinical trial. Clin. Exp. Dent. Res..

[94] Ibrahim S.S.A., Mandil I.A., Ezzatt O.M. (2024). Injectable platelet rich fibrin effect on laser depigmented gingiva: A clinical randomized controlled split mouth trial with histological assessment. J. Appl. Oral Sci..

[95] Kang M.J., Lee J.H., Hwang J., Chung S.H. (2023). Efficacy and safety of platelet-rich plasma and autologous-serum eye drops for dry eye in primary Sjogren’s syndrome: A randomized trial. Sci. Rep..

[96] Lahham C., Ta’a M.A., Lahham E., Michael S., Zarif W. (2023). The effect of recurrent application of concentrated platelet-rich fibrin inside the extraction socket on the hard and soft tissues. a randomized controlled trial. BMC Oral Health.

[97] Linnanmaki L., Kanto K., Karjalainen T., Leppanen O.V., Lehtinen J. (2020). Platelet-rich Plasma or Autologous Blood Do Not Reduce Pain or Improve Function in Patients with Lateral Epicondylitis: A Randomized Controlled Trial. Clin. Orthop. Relat. Res..

[98] Moftah N.H., Mansour A.M., Ibrahim S.M.A. (2022). Clinical evaluation of efficacy of intralesional platelet-rich plasma injection versus 1064 nm long-pulsed Neodymium:YAG laser in the treatment of inflammatory acne vulgaris in adolescent and post-adolescent patients: A prospective randomized split-face comparative study. Lasers Med. Sci..

[99] Navani A., Ambach M., Calodney A., Rosenthal R., Li G., Mahoney C.B., Everts P.A. (2024). The Safety and Effectiveness of Orthobiologic Injections for Discogenic Chronic Low Back Pain: A Multicenter Prospective, Crossover, Randomized Controlled Trial with 12 Months Follow-up. Pain Physician.

[100] Sharma R., Chaudhary N.K., Karki M., Sunuwar D.R., Singh D.R., Pradhan P.M.S., Gyawali P., Duwal Shrestha S.K., Bhandari K.K. (2023). Effect of platelet-rich plasma versus steroid injection in plantar fasciitis: A randomized clinical trial. BMC Musculoskelet. Disord..

[101] Shen M., Duan H., Lv R., Lv C. (2022). Efficacy of autologous platelet-rich plasma in preventing adhesion reformation following hysteroscopic adhesiolysis: A randomized controlled trial. Reprod. Biomed. Online.

[102] Shen Z., Zheng S., Chen G., Li D., Jiang Z., Li Y., Huang F. (2019). Efficacy and safety of platelet-rich plasma in treating cutaneous ulceration: A meta-analysis of randomized controlled trials. J. Cosmet. Dermatol..

[103] Singh S.K., Singh S. (2023). Effect of platelet counts and activator in platelet-rich plasma on the treatment of androgenetic alopecia, split-head comparison: A randomised, double-blind study. Indian J. Dermatol. Venereol. Leprol..

[104] Smith O.J., Leigh R., Kanapathy M., Macneal P., Jell G., Hachach-Haram N., Mann H., Mosahebi A. (2020). Fat grafting and platelet-rich plasma for the treatment of diabetic foot ulcers: A feasibility-randomised controlled trial. Int. Wound J..

[105] Sousa B.M., Lopez-Valverde N., Lopez-Valverde A., Caramelo F., Fraile J.F., Payo J.H., Rodrigues M.J. (2020). Different Treatments in Patients with Temporomandibular Joint Disorders: A Comparative Randomized Study. Medicina.

[106] Trull-Ahuir C., Sala D., Chismol-Abad J., Vila-Caballer M., Lison J.F. (2020). Efficacy of platelet-rich plasma as an adjuvant to surgical carpal ligament release: A prospective, randomized controlled clinical trial. Sci. Rep..

[107] Wei W., Zhang Y., Long B., Zhang Y., Zhang C., Zhang S. (2023). Injections of platelet-rich plasma prepared by automatic blood cell separator combined with topical 5% minoxidil in the treatment of male androgenetic alopecia. Skin Res. Technol..

[108] Wongjarupong A., Pairuchvej S., Laohapornsvan P., Kotheeranurak V., Jitpakdee K., Yeekian C., Chanplakorn P. (2023). “Platelet-Rich Plasma” epidural injection an emerging strategy in lumbar disc herniation: A Randomized Controlled Trial. BMC Musculoskelet. Disord..

[109] Xu Z., Wu S., Li X., Liu C., Fan S., Ma C. (2021). Ultrasound-Guided Transforaminal Injections of Platelet-Rich Plasma Compared with Steroid in Lumbar Disc Herniation: A Prospective, Randomized, Controlled Study. Neural Plast..

[110] Yavuz A., Gungormek H.S., Kuru L., Dogan B. (2024). Treatment of multiple adjacent gingival recessions using leucocyte- and platelet-rich fibrin with coronally advanced flap: A 12-month split-mouth controlled randomized clinical trial. Clin. Oral Investig..

[111] Zielinski M.A., Evans N.E., Bae H., Kamrava E., Calodney A., Remley K., Benyamin R., Franc D., Peterson M.R., Lovine J. (2022). Safety and Efficacy of Platelet Rich Plasma for Treatment of Lumbar Discogenic Pain: A Prospective, Multicenter, Randomized, Double-blind Study. Pain Physician.

[112] Amable P.R., Carias R.B., Teixeira M.V., da Cruz Pacheco I., Correa do Amaral R.J., Granjeiro J.M., Borojevic R. (2013). Platelet-rich plasma preparation for regenerative medicine: Optimization and quantification of cytokines and growth factors. Stem Cell. Res. Ther..

[113] Beitzel K., Allen D., Apostolakos J., Russell R.P., McCarthy M.B., Gallo G.J., Cote M.P., Mazzocca A.D. (2015). US definitions, current use, and FDA stance on use of platelet-rich plasma in sports medicine. J. Knee Surg..

[114] Kunze K.N., Pakanati J.J., Vadhera A.S., Polce E.M., Williams B.T., Parvaresh K.C., Chahla J. (2022). The Efficacy of Platelet-Rich Plasma for Ligament Injuries: A Systematic Review of Basic Science Literature With Protocol Quality Assessment. Orthop. J. Sports Med..

[115] Mazzocca A.D., McCarthy M.B., Chowaniec D.M., Cote M.P., Romeo A.A., Bradley J.P., Arciero R.A., Beitzel K. (2012). Platelet-rich plasma differs according to preparation method and human variability. J. Bone Jt. Surg. Am..

[116] Ramaswamy Reddy S.H., Reddy R., Babu N.C., Ashok G.N. (2018). Stem-cell therapy and platelet-rich plasma in regenerative medicines: A review on pros and cons of the technologies. J. Oral Maxillofac. Pathol..

[117] Sebbagh P., Cannone A., Gremion G., Gremeaux V., Raffoul W., Hirt-Burri N., Michetti M., Abdel-Sayed P., Laurent A., Warde N. (2023). Current Status of PRP Manufacturing Requirements & European Regulatory Frameworks: Practical Tools for the Appropriate Implementation of PRP Therapies in Musculoskeletal Regenerative Medicine. Bioengineering.

[118] Jildeh T.R., Su C.A., Vopat M.L., Brown J.R., Huard J. (2022). A Review of Commercially Available Point-of-Care Devices to Concentrate Platelet-Rich Plasma. Cureus.

[119] Kushida S., Kakudo N., Morimoto N., Hara T., Ogawa T., Mitsui T., Kusumoto K. (2014). Platelet and growth factor concentrations in activated platelet-rich plasma: A comparison of seven commercial separation systems. J. Artif. Organs.

[120] Kumar Thakur S.S., Kumar Negi D., Kaushik R., Kumar Ranjan D. (2019). Standardization of Platelet-Rich Plasma Preparation protocol: For desired and consistent composition of platelet. Int. J. Res. Anal. Rev..

[121] Page M.J., McKenzie J.E., Bossuyt P.M., Boutron I., Hoffmann T.C., Mulrow C.D., Shamseer L., Tetzlaff J.M., Akl E.A., Brennan S.E. (2021). The PRISMA 2020 statement: An updated guideline for reporting systematic reviews. J. Clin. Epidemiol..

[122] Higgins J.P.T., Thomas J., Chandler J., Cumpston M., Li T., Page M.J., Welch V.A. Cochrane Handbook for Systematic Reviews of Interventions Version 6.0. https://training.cochrane.org/handbook.

[123] Waskom M.L. (2021). seaborn: Statistical data visualization. J. Open Source Softw..

[124] Afzal G., Ahmed N., Zahoor F., Malik T., Farooq O. (2024). Efficacy of Platelet-Rich Plasma <em>versus</em> 5% Topical Monixidil for the Treatment of Androgenetic Alopecia. J. Coll. Physicians Surg. Pak..

[125] Arabaci O., Akyol M.E., Celikkaleli E., Sonmez B., Cetin E., Beger B. (2023). A randomized trial of the effects of platelet- rich plasma on postoperative complications after meningomyelocele sac repair. Eur. Rev. Med. Pharmacol. Sci..

[126] Efendieva Z., Vishnyakova P., Apolikhina I., Artemova D., Butov K., Kalinina E., Fedorova T., Tregubova A., Asaturova A., Fatkhudinov T. (2023). Hysteroscopic injections of autologous endometrial cells and platelet-rich plasma in patients with thin endometrium: A pilot randomized study. Sci. Rep..

[127] Kamble P., Prabhu R.M., Jogani A., Mohanty S.S., Panchal S., Dakhode S. (2023). Is Ultrasound (US)-Guided Platelet-Rich Plasma Injection More Efficacious as a Treatment Modality for Lateral Elbow Tendinopathy Than US-Guided Steroid Injection?: A Prospective Triple-Blinded Study with Midterm Follow-up. Clin. Orthop. Surg..

[128] Chuah S.Y., Tan C.H., Wang E.C.E., Tan K.T., Chan R.K.W., Zhao X., Lee S.S.J. (2023). Efficacy of platelet-rich plasma in Asians with androgenetic alopecia: A randomized controlled trial. Indian J. Dermatol. Venereol. Leprol..

[129] Kotb S.Y., Sherif N.M., Saleh H.A., Ahmed S.F., Sakr H.M., Taeimah M.O. (2022). The role of intra-articular injection of autologous platelet-rich plasma versus corticosteroids in the treatment of synovitis in lumbar facet joint disease. Saudi Med. J..

[130] Metheetrairut C., Ngowyutagon P., Tunganuntarat A., Khowawisetsut L., Kittisares K., Prabhasawat P. (2022). Comparison of epitheliotrophic factors in platelet-rich plasma versus autologous serum and their treatment efficacy in dry eye disease. Sci. Rep..

[131] Won S.J., Kim D.Y., Kim J.M. (2022). Effect of platelet-rich plasma injections for chronic nonspecific low back pain: A randomized controlled study. Medicine.

[132] Breton A., Leplat C., Picot M.C., Aouinti S., Taourel P., Laffont I., Julia M., Cyteval C. (2022). Prediction of clinical response to corticosteroid or platelet-rich plasma injection in plantar fasciitis with MRI: A prospective, randomized, double-blinded study. Diagn. Interv. Imaging.

[133] Shah S.A., Singh B.P., Rao J., Kumar L., Singh M., Singh P.K. (2021). Biological and esthetic outcome of immediate dental implant with the adjunct pretreatment of immediate implants with platelet-rich plasma or photofunctionalization: A randomized controlled trial. J. Indian Prosthodont. Soc..

[134] Aghajanova L., Sundaram V., Kao C.N., Letourneau J.M., Manvelyan E., Cedars M.I., Huddleston H.G. (2021). Autologous platelet-rich plasma treatment for moderate-severe Asherman syndrome: The first experience. J. Assist. Reprod. Genet..

[135] Bakhsh A.S., Maleki N., Sadeghi M.R., SadeghiTabar A., Tavakoli M., Zafardoust S., Karimi A., Askari S., Jouhari S., Mohammadzadeh A. (2022). Effects of Autologous Platelet-Rich Plasma in women with repeated implantation failure undergoing assisted reproduction. JBRA Assist. Reprod..

[136] Hersant B., SidAhmed-Mezi M., Aboud C., Niddam J., Levy S., Mernier T., La Padula S., Meningaud J.P. (2021). Synergistic Effects of Autologous Platelet-Rich Plasma and Hyaluronic Acid Injections on Facial Skin Rejuvenation. Aesthet. Surg. J..

[137] Kuo S.J., Chou W.Y., Hsu C.C., Chang-Chien G.P., Lin S.F., Siu K.K., Tsai T.C., Ko J.Y., Sun Y.C. (2020). Systemic effects of platelet-rich plasma local injection on serum and urinary anabolic metabolites: A prospective randomized study. Chin. J. Physiol..

[138] El-Timamy A., El Sharaby F., Eid F., El Dakroury A., Mostafa Y., Shaker O. (2020). Effect of platelet-rich plasma on the rate of orthodontic tooth movement. Angle Orthod..

[139] Ragab S.E.M., Nassar S.O., Morad H.A., Hegab D.S. (2020). Platelet-rich plasma in alopecia areata: Intradermal injection versus topical application with transepidermal delivery via either fractional carbon dioxide laser or microneedling. Acta Dermatovenerol. Alp. Pannonica Adriat..

[140] Thu A.C., Kwak S.G., Shein W.N., Htun M., Htwe T.T.H., Chang M.C. (2020). Comparison of ultrasound-guided platelet-rich plasma injection and conventional physical therapy for management of adhesive capsulitis: A randomized trial. J. Int. Med. Res..

[141] Pakhomova E.E., Smirnova I.O. (2020). Comparative Evaluation of the Clinical Efficacy of PRP-Therapy, Minoxidil, and Their Combination with Immunohistochemical Study of the Dynamics of Cell Proliferation in the Treatment of Men with Androgenetic Alopecia. Int. J. Mol. Sci..

[142] Wu S., Lin W., Xu W., Li H. (2020). Clinical study on reconstruction of posterior cruciate ligament with platelet rich plasma combined with 3-strand peroneus longus tendons. Zhongguo Xiu Fu Chong Jian Wai Ke Za Zhi.

[143] Babu N., Kohli P., Ramachandran N.O., Adenuga O.O., Ahuja A., Ramasamy K. (2020). Comparison of platelet-rich plasma and inverted internal limiting membrane flap for the management of large macular holes: A pilot study. Indian J. Ophthalmol..

[144] Saha S., Patra A.C., Gowda S.P., Mondal N., Rahaman S., Ahmed S.K.S., Debbarma S., Vitthal K.P.K., Sarkar S., Sil A. (2020). Effectiveness and safety of autologous platelet-rich plasma therapy with total contact casting versus total contact casting alone in treatment of trophic ulcer in leprosy: An observer-blind, randomized controlled trial. Indian J. Dermatol. Venereol. Leprol..

[145] Kawabata S., Hachiya K., Nagai S., Takeda H., Rashid M.Z.M., Ikeda D., Kawano Y., Kaneko S., Ohno Y., Fujita N. (2023). Autologous Platelet-Rich Plasma Administration on the Intervertebral Disc in Low Back Pain Patients with Modic Type 1 Change: Report of Two Cases. Medicina.

[146] Pretorius J., Habash M., Ghobrial B., Alnajjar R., Ellanti P. (2023). Current Status and Advancements in Platelet-Rich Plasma Therapy. Cureus.

[147] Miron R., Choukroun J., Ghanaati S. (2018). Controversies related to scientific report describing g-forces from studies on platelet-rich fibrin: Necessity for standardization of relative centrifugal force values. Int. J. Growth Factors Stem Cells Dent..

[148] Irmak G., Demirtas T.T., Gumusderelioglu M. (2020). Sustained release of growth factors from photoactivated platelet rich plasma (PRP). Eur. J. Pharm. Biopharm..

[149] Everts P., Onishi K., Jayaram P., Lana J.F., Mautner K. (2020). Platelet-Rich Plasma: New Performance Understandings and Therapeutic Considerations in 2020. Int. J. Mol. Sci..

[150] Pietrzak W.S., Eppley B.L. (2005). Platelet rich plasma: Biology and new technology. J. Craniofac Surg..

[151] FDA Additional Standards for Human Blood and Blood Products. https://www.accessdata.fda.gov/scripts/cdrh/cfdocs/cfcfr/CFRSearch.cfm?CFRPart=640.

[152] Marx R.E., Carlson E.R., Eichstaedt R.M., Schimmele S.R., Strauss J.E., Georgeff K.R. (1998). Platelet-rich plasma: Growth factor enhancement for bone grafts. Oral Surg. Oral Med. Oral Pathol. Oral Radiol. Endodontology.

[153] Marx R.E. (2004). Platelet-rich plasma: Evidence to support its use. J. Oral Maxillofac. Surg..

[154] ICMS.Platelet Rich Plasma (PRP) Guidelines. http://www.cellmedicinesociety.org/attachments/370_Section%2010%20-%20Platelet%20Rich%20Plasma%20(PRP)%20Guidelines.pdf.

[155] EEA Commission Directive 2005/62/EC. https://eur-lex.europa.eu/LexUriServ/LexUriServ.do?uri=OJ:L:2005:256:0041:0048:EN:PDF.

[156] DHA Standards for Platelet Rich Plasma Therapy. https://dhcc.ae/gallery/Standards%20for%20Platelet%20Rich%20Plasma%20(PRP)%20Services.pdf.

[157] Elphingstone J.W., Alston E.T., Colorado B.S. (2024). Platelet-rich plasma for nonoperative management of degenerative meniscal tears: A systematic review. J. Orthop..

[158] Pelaez-Gorrea P., Damia-Gimenez E., Rubio-Zaragoza M., Cuervo-Serrato B., Hernandez-Guerra A.M., Miguel-Pastor L., Del Romero-Martinez A., Sopena-Juncosa J., Torres-Torrillas M., Santana A. (2023). The autologous chondral platelet-rich plasma matrix implantation. A new therapy in cartilage repair and regeneration: Macroscopic and biomechanical study in an experimental sheep model. Front. Vet. Sci..

[159] Howlader M.A.A., Almigdad A., Urmi J.F., Ibrahim H. (2023). Efficacy and Safety of Hyaluronic Acid and Platelet-Rich Plasma Combination Therapy Versus Platelet-Rich Plasma Alone in Treating Knee Osteoarthritis: A Systematic Review. Cureus.

[160] Nie L.Y., Zhao K., Ruan J., Xue J. (2021). Effectiveness of Platelet-Rich Plasma in the Treatment of Knee Osteoarthritis: A Meta-analysis of Randomized Controlled Clinical Trials. Orthop. J. Sports Med..

[161] Jawanda H., Khan Z.A., Warrier A.A., Acuna A.J., Allahabadi S., Kaplan D.J., Ritz E., Jackson G.R., Mameri E.S., Batra A. (2024). Platelet-Rich Plasma, Bone Marrow Aspirate Concentrate, and Hyaluronic Acid Injections Outperform Corticosteroids in Pain and Function Scores at a Minimum of 6 Months as Intra-Articular Injections for Knee Osteoarthritis: A Systematic Review and Network Meta-analysis. Arthroscopy.

