# Supplementary material for: Systematic Review of Platelet-Rich Plasma in Medical and Surgical Specialties: Quality, Evaluation, Evidence, and Enforcement
Source: J Clin Med. 2024 Aug 5;13(15):4571. doi: 10.3390/jcm13154571 (PMC11313071; doi:10.3390/jcm13154571)
Supplement: Supplementary file 1 [file jcm-13-04571-s001.zip › jcm-3092345-Supplementary Material S1 Search strategy.pdf]

**PubMed Search Strategy:**

1. **Keywords and MeSH terms:**
    - "Platelet-Rich Plasma" OR "PRP" OR "Autologous Platelet Gel" OR "Platelet Concentrate"
    - "Aesthetic Medicine" OR "Cosmetic Dermatology" OR "Facial Rejuvenation" OR "Skin Rejuvenation" OR "Hair Restoration" OR "Alopecia Treatment" OR "Anti-Aging" OR "Dermal Fillers"
    - "Regenerative Medicine" OR "Tissue Regeneration" OR "Wound Healing" OR "Osteoarthritis Treatment" OR "Surgical Procedures" OR "Tendon Repair" OR "Ligament Healing" OR "Cartilage Repair" OR "Musculoskeletal Injuries" OR "Infertility" OR "Clinical Medicine"
    - "Efficacy" OR "Safety" OR "Adverse Effects"
    - "Quality Control" OR "Standardization" OR "Regulatory Approval"
    - "Observational Studies" OR "Randomized Controlled Trials"
2. **Boolean Operators:**
    - Combine the above terms using AND/OR.

**EMBASE Search Strategy:**

1. **Keywords and Emtree terms:**
    - 'Platelet Rich Plasma'/exp OR 'PRP' OR 'Autologous Platelet Gel' OR 'Platelet Concentrate'

- 'Aesthetic Medicine' OR 'Cosmetic Dermatology' OR 'Facial Rejuvenation' OR 'Skin Rejuvenation' OR 'Hair Restoration' OR 'Alopecia Treatment' OR 'Anti-Aging' OR 'Dermal Fillers'
- 'Regenerative Medicine' OR 'Tissue Regeneration' OR 'Wound Healing' OR 'Osteoarthritis Treatment' OR 'Surgical Procedures' OR 'Tendon Repair' OR 'Ligament Healing' OR 'Cartilage Repair' OR 'Musculoskeletal Injuries' OR 'Infertility' OR 'Clinical Medicine'
- 'Efficacy' OR 'Safety' OR 'Adverse Effects'
- 'Quality Control' OR 'Standardization' OR 'Regulatory Approval'
- 'Observational Studies' OR 'Randomized Controlled Trials'

2. **Boolean Operators:**
   - Combine the above terms using AND/OR.

**Web of Science Search Strategy:**

1. **Keywords:**
   - "Platelet-Rich Plasma" OR "PRP" OR "Autologous Platelet Gel" OR "Platelet Concentrate"
   - "Aesthetic Medicine" OR "Cosmetic Dermatology" OR "Facial Rejuvenation" OR "Skin Rejuvenation" OR "Hair Restoration" OR "Alopecia Treatment" OR "Anti-Aging" OR "Dermal Fillers"
   - "Regenerative Medicine" OR "Tissue Regeneration" OR "Wound Healing" OR "Osteoarthritis Treatment" OR "Surgical Procedures" OR

"Tendon Repair" OR "Ligament Healing" OR "Cartilage Repair" OR "Musculoskeletal Injuries" OR "Infertility" OR "Clinical Medicine"

- "Efficacy" OR "Safety" OR "Adverse Effects"
- "Quality Control" OR "Standardization" OR "Regulatory Approval"
- "Observational Studies" OR "Randomized Controlled Trials"

2. **Boolean Operators:**

- Combine the above terms using AND/OR.
